# Synthesis of γ-hydroxypropyl P-chirogenic (±)-phosphorus oxide derivatives by regioselective ring-opening of oxaphospholane 2-oxide precursors

**DOI:** 10.3762/bjoc.11.143

**Published:** 2015-07-30

**Authors:** Iris Binyamin, Shoval Meidan-Shani, Nissan Ashkenazi

**Affiliations:** 1 Department of Organic Chemistry, IIBR-Israel Institute for Biological Research, P.O. Box 19, Ness Ziona, 74100, Israel

**Keywords:** chirogenic phosphorus, Grignard reagents, oxaphospholanes, phosphinates, phosphine oxides

## Abstract

The synthesis of P-chirogenic (±)-phosphine oxides and phosphinates via selective nucleophilic ring opening of the corresponding oxaphospholanes is described. Two representative substrates: the phosphonate 2-ethoxy-1,2-oxaphospholane 2-oxide and the phosphinate 2-phenyl-1,2-oxaphospholane 2-oxide were reacted with various Grignard reagents to produce a single alkyl/aryl product. These products may possess further functionalities in addition to the phosphorus center such as the γ-hydroxypropyl group which results from the ring opening and π-donor moieties such as aryl, allyl, propargyl and allene which originates from the Grignard reagent.

## Introduction

Organophosphorus compounds containing phosphorus to carbon bond(s) are widely used in organic transformations. Textbook examples are the Wittig and the Horner–Wadsworth–Emmons reactions. Moreover, the vast majority of ligands used in organometallic catalysis possess this bond(s). Among which organophosphorus compounds bearing three different alkyl (or aryl) ligands are of a great interest, as such compounds possess the essential backbone for P-chirogenic derivatives [1–3]. The formation of P–C bonds [4–6] may be classified into two main types: (A) Attack by nucleophilic low valent phosphorus compounds (P(III) or phosphide ions P(II)) on positively charged carbon centers (e.g., Michaelis–Arbuzov [7], Michaelis–Becker [8], Pudovik [9], Friedel–Crafts reactions [10], catalytic hydrophosphorylation [11] and others). Notably, these reactions commonly result in the formation of a pentavalent P-center which can no longer be exploited as a nucleophile. Therefore, this type of reaction can be used for the introduction of only one alkyl ligand. (B) Electrophilic P(V) compounds which can undergo a nucleophilic attack by various carbanions to form new P(V)-carbon bond(s) [12]. While the former synthetic approach has been studied extensively and its scope and limitations are well documented, the full potential of the latter approach may be regarded as only partially exploited.

Similar to Grignard reactions with carboxylic esters, the formation of a P–C bond from phosphate esters using these reagents, is limited [13]. The reactions are not selective and compounds containing multiple P–C bonds are generally obtained [14]. In order to achieve better selectivity, a single alkyloxy substituent may be replaced by a halogen. This halogen will be substituted selectively under milder Grignard conditions to form a single P–C bond, leaving the other ester groups intact [15–16].

**Figure 1:**
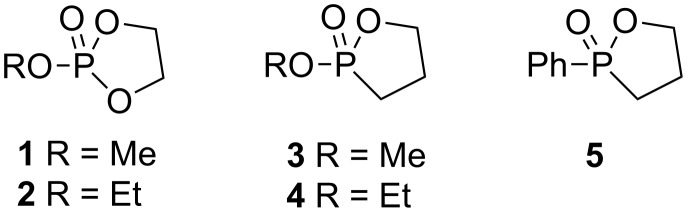
Chemical structures of 2-methoxy-1,3,2-dioxaphospholane 2-oxide (**1**), 2-ethoxy-1,3,2-dioxaphospholane 2-oxide (**2**), 2-methoxy-1,2-oxaphospholane 2-oxide (**3**), 2-ethoxy-1,2-oxaphospholane 2-oxide (**4**), and 2-phenyl-1,2-oxaphospholane 2-oxide (**5**).

**Scheme 1:**
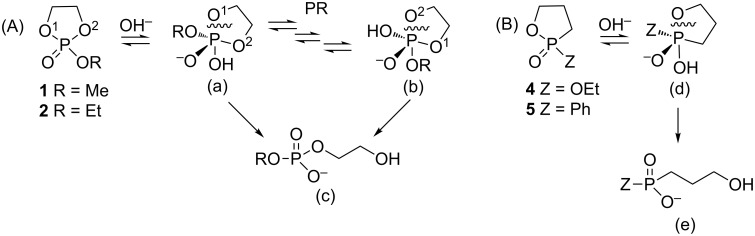
(A) Alkaline hydrolysis of dioxaphospholane: the phosphorane intermediate includes one endocyclic oxygen atom at the apical position (a). Pseudorotation(s) may direct to a more stable TBP intermediate (b) [28]. Thus, the final product (c) can result from either P–O^1^ or P–O^2^ cleavage. (B) Alkaline hydrolysis of oxaphospholane: the most stable phosphorane intermediate in which the endocyclic oxygen atom is at the apical position (d) will not undergo pseudorotation(s) due to a high energetic barrier. The ring opening to the final product (e) is therefore regioselective.

Cyclic phosphorus diesters are much more reactive towards nucleophilic reagents than their acyclic analogs. Specifically, 5-membered cyclic esters of phosphonic and phosphoric acids are known to undergo acid and alkaline hydrolysis at rates 10^5^–10^8^ times faster than their acyclic analogs [17]. Thus, cyclic esters may be regarded as a halogen equivalent in terms of their leaving group ability under nucleophilic displacement conditions [18]. The rapid hydrolysis of 2-methoxy-1,3,2-dioxaphospholane 2-oxide (**1**, Figure 1) exclusively forms a ring opening product [19–21]. This reaction proceeds via a trigonal bipyramidal (TBP) intermediate where the ring is constrained to span on apical and equatorial positions. Endocyclic cleavage of the phosphorus–oxygen bond at the apical position produces β-hydroxyethylphosphate (Scheme 1A). Dioxaphospholane **1** contains two identical oxygen atoms that can be cleaved with no preference due to fast pseudorotation between two practically energetically equivalent phosphorane intermediates, in which these two oxygen centers interconvert between apical to equatorial positions (a and b) [20]. Notably, some selectivity could be obtained once the P center of the phospholane is ligated to two different heteroatoms (i.e., O, N or O, S) [22–27], albeit, both the P–O and the P–N bonds are susceptible to cleavage.

Kinetic studies of the ring openings of oxaphospholanes under acidic or alkaline conditions were also reported. 2-Methoxy-1,2-oxaphospholane 2-oxide (**3**) [29] (Figure 1) undergoes a P–O cleavage two orders of magnitude slower than the dioxaphospholane **1** [17], but at least three orders of magnitude faster than its acyclic analogs. Similarly, 2-phenyl-1,2-oxaphospholane 2-oxide (**5**) [30–31] (Figure 1) hydrolyzed under basic conditions 2 orders of magnitude slower than the dioxaphospholane analog, but 6.2 × 10^3^ faster than the corresponding acyclic analog [32].

The ring opening of **3** and **5** under hydrolytic conditions was found to be completely regioselective [20], since the P–C bond occupies the equatorial position according to the preference rules [33]. Therefore, fast pseudorotations (PR), either Berry’s pseudorotation or Turnstile rotation [34], which form identical TBP intermediates, are suppressed (Scheme 1B). Thus, oxaphospholanes can potentially form a new P–C bond in an entirely regioselective manner via cleavage of the single endocyclic P–O bond [35–37]. Based on this mechanism, the unique properties of oxaphosphorinanes and oxaphospholanes may also be used to form chirogenic (±)-phosphorus compounds [26,38–39].

**Scheme 2:**
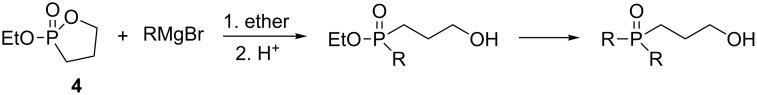
Reaction of **4** with various Grignard reagents.

In our previous work, we investigated the reaction of 2-ethoxy-1,3,2-dioxaphospholane 2-oxide (**2**) with Grignard reagents, and showed that phosphonates were formed as single products via a regioselective endocyclic P–O bond cleavage [40–42]. In continuation of this work, we envisaged to expand this regioselective ring-opening strategy to oxaphospholane precursors in order to afford P-chirogenic (±)-phosphine oxide and phosphinate derivatives (Scheme 2).

Herein, we disclose our results on the reactions of oxaphospholanes 2-ethoxy-1,2-oxaphospholane 2-oxide (**4**) and **5** with various Grignard reagents. Indeed, in all cases, the attack on the phosphorus atom led exclusively to ring opening via endocyclic P–O bond cleavage and formation of a new P–C bond, yielding phosphinates and phosphine oxides with three different substituents on the phosphorus atom.

## Results and Discussion

In order to establish that under Grignard conditions the selectivity between the endocyclic and the exocyclic P–O bonds is not unique to **1** and **2**, and that it is also retained in other oxaphospholanes, we first ran a series of reactions using the well-known oxaphospholane **4** [43] with various Grignard reagents such as aliphatic (Me, Et), aromatic (Ph), cyclic (cyclopentyl) or allylic derivativess (Table 1). The products were identified by multinuclear NMR and MS (CI) detection.

**Table 1:** Synthesis of γ-hydroxypropyl (±)-phosphinates^a^ and related compounds.

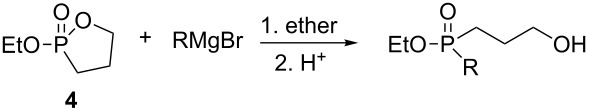				

Entry	R	Product	% yield (isolated)	^31^P NMR (δ)

1	Me	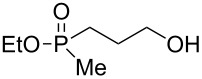 **4a**	73 (68)	+53.5
2	Et	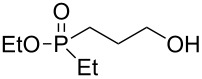 **4b**	67 (62)	+57.3
3^b^	All	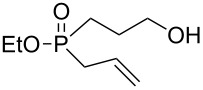 **4c**	58 (50)	+51.8
4	c-Pent	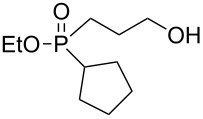 **4d**	76 (30)	+58.4
5^c^	Ph	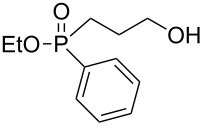 **4e**	85 (38)	+43.1
		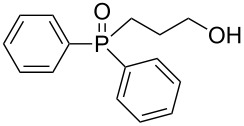 **5e**	10	+32.1

^a^Reagents and conditions: 0.5 mmol of **4** and 1.5 mmol of the Grignard reagent were reacted in dry ether (5 mL) at 0 °C, and stirred at rt for 0.5–1 h. At the end of the reaction, the compound was hydrolyzed with 1.5 mmol 1 N HCl/ether. ^b^4 equiv of allylMgBr were used. ^c^2 equiv of PhMgBr were used.

Generally, the reaction was performed in cooled (0 ºC) ether, using 3 equiv of the Grignard reagent, while warming to rt with stirring. The reaction time was determined using ^31^P NMR monitoring, and we found that indeed, the attack of the Grignard reagents on the phosphorus atom of **4** led to an exclusive ring opening and the formation of the corresponding phosphinates within 0.5–1 hour. We noticed that prolonged stirring at rt did not increase the conversion percentage to the desired products significantly. Under these reaction conditions 58–85% yield of **4a–e** were observed and the pure products were isolated in 30–80% yield following flash chromatography. The relative amount of the Grignard reagents used was found to be crucial as one equiv led to an incomplete reaction, while more than 3 equiv of the Grignard reagent led to dialkylation of the phosphorus atom, and formation of phosphine oxide side products. This phenomenon was most noticeable using phenylmagnesium bromide as a nucleophile: 2 equiv of the reagent were required to form **4e** in 85% yield. The side product **5e**, which arises from side reaction of **4e** with PhMgBr (Table 2), was obtained in 10% yield. In the case of the allyl compound, we increased the amount of allylmagnesium bromide up to 4 equivalents; however, the yield of **4c** was limited to 58%. The same trend was observed in our previous study once **2** was reacted with allylmagnesium chloride to form the respective allylphosphonate in considerably low yield [40–42].

The measured ^31^P NMR chemical shifts of **4a–d** appear at the characteristic region of 51–58 ppm, whereas for **4e**, which contains a highly electronegative benzene ring, the shift appears at 43 ppm. The ^13^C NMR is most indicative for these systems. The signal of the propyl carbon in the α-possition to the phosphorus atom in compounds **4a–e** was found to be at ~25 ppm with a doublet split pattern (*J*_P-C_ average of 90 Hz). Another doublet with a similar *J*_P-C_ coupling exist for the carbon of the R group. The chemical shift of this signal changes as a function of the R group character. The MS (CI) for all the phosphinates showed the *m*/*z* [M + 1] signal.

**Scheme 3:**
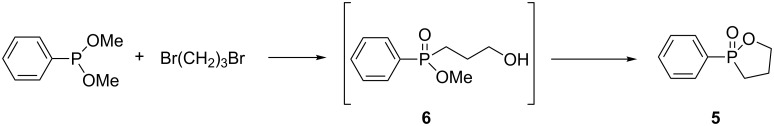
Synthesis of 2-phenyl-1,2-oxaphospholane 2-oxide (**5**).

It is well established that phosphinates can be readily converted into phosphine oxides at somewhat elevated temperatures [44–45]. Our hypothesis was that another possible attractive route to obtain phosphine oxides would be the reaction of aryl/alkyl-oxaphospholanes with Grignard reagents. Oxaphospholane **5** was obtained according to Garner’s procedure [43] using a one pot synthesis from two commercial starting materials, without isolation of intermediate **6** (Scheme 3). This method was found to be superior to two other protocols: reaction of phenyl disulfide with 3-hydroxypropyl(phenyl)phosphine oxide [30] and reaction of allyl alcohol with benzene phosphinic acid monobutyl ester in the presence of di-*tert*-butyl peroxide [46]. As both methods utilize phosphorus starting materials which are not commercially available and a multistep synthesis is required for their preparation.

As with oxaphospholane **4**, compound **5** was reacted with 3 equiv of various Grignard reagents to produce phosphine oxides in high yields (83–97%), leading after work-up and flash chromatography purification to high to moderate (38–95%) isolated yields (Table 2). In general, the relatively higher yields of the phosphine oxides **5a–5e** compared to the phosphinate analogs **4a–e** can be attributed to the lack of the exocyclic ester group in **5**, which may be susceptible to a second alkylation/arylation.

**Table 2:** Synthesis of γ-hydroxypropyl (±)-phosphine oxides^a^.

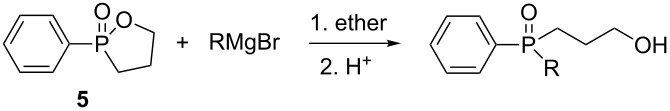				

Entry	R	(**5**)	% Yield (isolated)	^31^P NMR (δ)

1	Me	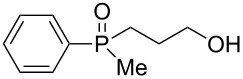 **5a**	90 (77)	+37.5
2	Et	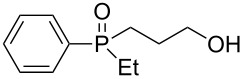 **5b**	83 (79)	+41.9
3^b^	All	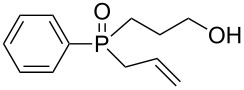 **5c**	87 (71)	+37.1
4	c-Pent	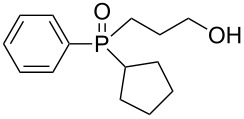 **5d**	91 (62)	+42.8
5	Ph	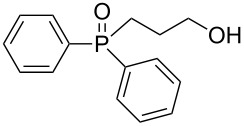 **5e**	97 (95)	+32.2

^a^Reagents and conditions: 0.5 mmol of **5** and 1.5 mmol of the Grignard reagent were reacted in dry ether (5 mL) at 0 °C, and stirred to rt for 1 h. At the end of the reaction the compound was hydrolyzed with 1.5 mmol 1 N HCl/ether. ^b^4 equiv of allylMgBr were used.

The ^31^P NMR chemical shifts of **5a–d** appear at the characteristic ~+40 ppm region. The respective signal for **5e**, which possess a second aryl substituent, appears in a higher field of δ +32 ppm, as expected. Most indicative and informative are the ^13^C NMR spectra. In comparison to the phosphinates, the ^13^C NMR showed an average *J*_P-C_ of 95 Hz for the phenyl quaternary carbon at +132 ppm. The *J*_P-C_ of the carbons were smaller than in the phosphinate system: *J*_P-C_ of 65–73 Hz for the propyl carbon, and *J*_P-C_ of 70 Hz for the R group (except for R = cyclopentyl, *J*_P-C_ = 40 Hz). These results are in accordance to those of Simonnin et al. who showed that increasing the number of alkyl substituents on a phosphorus atom results in decrease of the *J*_P-C_ values [47].

**Scheme 4:**
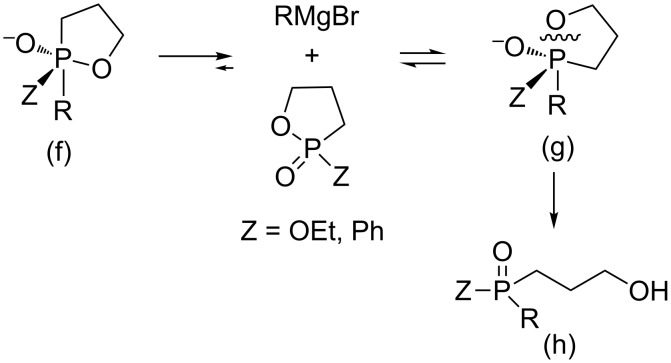
Formation of phosphinates and phosphine oxides bearing three different substituents from oxaphospholane and Grignard reagents: The phosphorane intermediate includes an oxygen atom at the apical position (g) and does not undergo pseudorotation to form the methylene group at the apical position due to an energetic barrier. The final product (h) is therefore formed regioselectivly.

All these results indeed indicate that during the reactions a pentavalent TBP phosphorane intermediate is formed, which could not undergo pseudorotation due to a high energetic barrier. The endocyclic P–O bond is cleaved almost exclusively (Scheme 4) to form the respective phosphinates and phosphine oxides in high yields.

**Scheme 5:**
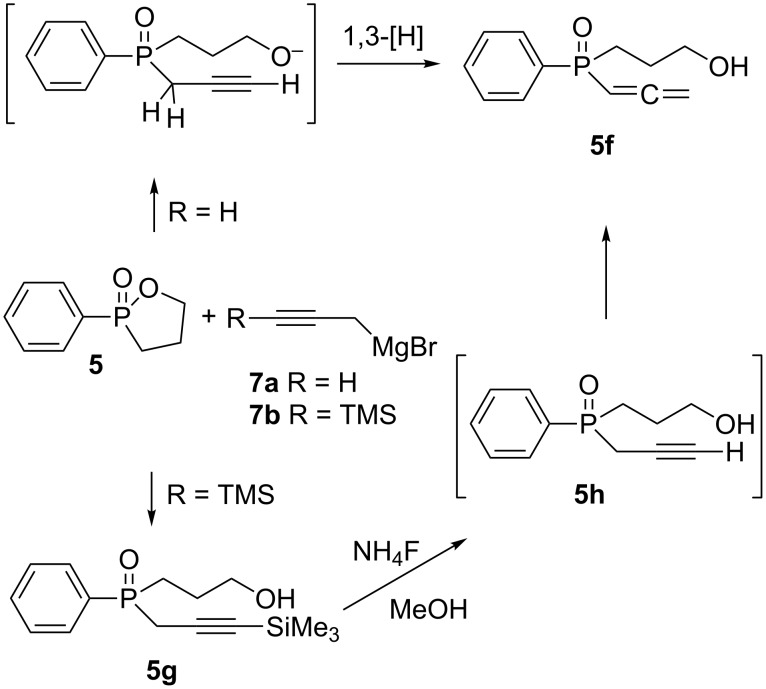
Synthesis of acetylene and allene phosphine oxides.

Propargyl organophosphorus compounds present a unique interest from a synthetic point of view due to their ability to create new phosphines [48] or to be involved in various reactions (e.g., [2 + 3] cycloaddition) [49–50]. Moreover, propargyl compounds are known to undergo base catalyzed 1,3-prototropic rearrangments to the corresponding allenes [51], a highly usefull class of compounds [52–54]. Thus, we attempted to produce a propargylic analog by reacting **5** with propargylmagnesium bromide. The conditions required for this reaction were harsher than the previous ones: 5 equiv of the Grignard reagent and **5** were heated in THF for 18 h. Further heating did not provide higher conversion. Following hydrolytic work-up and flash chromatographic purification the product was identified as allene **5f**, rather than acetylene **5h**, due to propargyl–allene rearrangement (Scheme 5). Most characteristic are the ^13^C NMR chemical shift of HC=*C*=CR at 213.36 ppm and the ^1^H NMR signals at 5.65 ppm (dt, *J*_H1-H2_ = 6.9 Hz, *J*_H1-P_ = 2.7 Hz) and 5.05 ppm (dd, *J*_H2-P_ = 10.8 Hz, *J*_H2-H1_ = 6.9 Hz) which, together with the ^31^P chemical shift at +28.33 ppm, are in excellent agreement with the data reported in the literature for similar systems [55]. The expected mass (identical in the case of **5f** and **5h**) was also obtained by MS (CI). Thus, it is likely to asume that the basic conditions favorize the deprotonation of a proton from the α-possition to both the phosphoryl and the propargyl moieties, to afford the allenyl derivative by isomerization. Noteably, we cannot exclude the possibility that this rearrangement occurs already on the Grignard reagent and that the actual nucleophile is allenemagnesium bromide [56–57]. Nevertheless, it is reasonable to assume that prior to its acidic work-up **5h** contains an intramolecular alkoxide function that may certainly serve as the catalytic base that prompts the rearrangment.

The 1,3-hydrogen shift in a P-propargyl system is well established [51]. In an attempt to prevent this rearrangement we applied an acetylene substituted with the bulky TMS group [58], which may be easily removed at a later stage, and formed the 3-(trimethylsilyl)propargylmagnesium bromide (**7b**) [59]. The reaction of **5** with 5 equiv of **7b** in THF for 48 h produced the protected propargylic analog **5g** in 35% isolated yield. The ^31^P NMR of this product exhibited a signal at +35.6 ppm, and the ^13^C NMR chemical shifts of the two acetylenic carbons were observed at 97.12 ppm (d, *J* = 9.0 Hz) and 89.80 ppm (d, *J* = 7.5 Hz). Attempts to cleave the TMS protecting group [60] of **5g** led to the rearrangement product **5f**. Therefore, if a non-substituted porpargyl moiety is desired the TMS removal should be performed at a later stage of the synthesis (i.e., after the "click" reaction) [61].

## Conclusion

In this work, we demonstrated the generality and the versatility of oxaphospholanes such as **4** and **5** to serve as precursors for the synthesis of γ-hydroxypropyl (±)-phosphinates and phosphine oxides, respectively, using Grignard reactions. Noteworthy is the fact that the γ-hydroxypropyl substituent, which results directly from the ring opening of the phospholane, could provide a second ligation site upon complexation of the corresponding phosphines to a transition metal catalyst. These P,O bidentate ligands are quite useful in organometallic compounds once binded to metals which favor a six-membered ring geometry. Moreover, these compounds could be advantageously used in organocatalysis and are commonly embedded in inorganic or organic matrix for various heterogeneous applications.

## Supporting Information

File 1 Experimental details, characterization data and ^1^H and ^13^C NMR spectra of all new compounds.
