# Supplementary material for: Synthesis of γ-hydroxypropyl P-chirogenic (±)-phosphorus oxide derivatives by regioselective ring-opening of oxaphospholane 2-oxide precursors
Source: Beilstein J Org Chem. 2015 Jul 30;11:1332–9. doi: 10.3762/bjoc.11.143 (PMC4578417; doi:10.3762/bjoc.11.143)
Supplement: File 1 — Experimental details, characterization data and 1H and 13C NMR spectra of all new compounds. [file Beilstein_J_Org_Chem-11-1332-s001.pdf]

Supporting Information

for

Synthesis of γ-hydroxypropyl P-chirogenic (±)-phosphorus oxide derivatives by regioselective ring-opening of oxaphospholane 2-oxide precursors

Iris Binyamin, Shoval Meidan-Shani and Nissan Ashkenazi*

Address: Department of Organic Chemistry, IIBR-Israel Institute for Biological Research, P.O. Box 19, Ness Ziona, 74100, Israel

Email: Nissan Ashkenazi - nissan.ashkenazi@iibr.gov.il

* Corresponding author

Experimental details, characterization data and ^{1}H and ^{13}C NMR spectra of all new compounds

Table of Contents

General Information S2

General experimental procedure and spectral data for all compounds S2–S8

^{1}H and ^{13}C NMR charts of compounds S9–S19

References S20

General information

All commercially available reagents were used without further purification. The concentrations of the Grignard reagents were determined prior to usage. Compound **7** was synthesized similar to a published procedure [1] from 3-(trimethylsilyl)propargyl bromide. Compound **4** was prepared from diethyl (3-bromopropyl)phosphonite [2] according to the published procedure [3,4].

All solvents were dried before use by standard methods and stored under nitrogen. All reactions were performed under nitrogen atmosphere. Flash column chromatography was performed using silica gel 60 (0.040–0.063 mm). Nuclear magnetic resonance (NMR) spectra were recorded on Advance Bruker 300 and 500 spectrometers. Mass spectra (CI) were recorded on a Varian (Saturn 2200) instrument.

2-Phenyl-1,2-oxaphospholane 2-oxide (5):

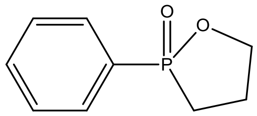

2-Phenyl-1,2-oxaphospholane 2-oxide was prepared by a modification of the procedure that was reported by Garner [1]. Thus, dimethyl(phenyl)phosphonite (6.0 g, 0.035 mol) and 1,3-dibromopropane (35.6 g, 0.17 mol) were placed in a flask fitted with a reflux condenser and heated to 150 °C (external temperature) for 2 hours, under N_2 flow. The excess of 1,3-dibromopropane was distilled off and the product was isolated by flash chromatography ($CHCl_3$) to afford 1.34 g of **4** (21%).

^{1}H ($CDCl_3$, 300 MHz): δ 7.87-7.78 (m, 2H), 7.64-7.56 (m, 1H), 7.55-7.46 (m, 2H) 4.66 (m, 1H), 4.48-4.46 (m, 1H), 2.52-2.21 (m, 3H), 2.07-1.95 (m, 1H); ^{13}C ($CDCl_3$, 75.5 MHz): δ 132.62 (d, $J_{P\text{-}C}$= 11.7 Hz), 131.49 (d, $J_{P\text{-}C}$= 10.6 Hz), 128.67 (d, $J_{P\text{-}C}$= 13.2 Hz), 70.60 (d, $J_{P\text{-}C}$= 4.8 Hz), 25.68 (d, $J_{P\text{-}C}$= 82.5 Hz), 24.50; ^{31}P {^{1}H} NMR ($CDCl_3$, 121.5 MHz) δ 54.46. The quaternary carbon of the phenyl ring was not detected.

GCMS-CI (m/z) 183 (100%) $(M+1)^+$, 141 (86%) (PhP(O)OH).

General experimental procedure

To a dried three neck flask equipped with a separating funnel, nitrogen inlet and a condenser with nitrogen outlet, **4** or **5** (0.5 mmol) in anhydrous ether (5 mL) was placed. The solution was cooled in an ice bath and the Grignard reagent (1.5 mmol) was added dropwise, to form a precipitation. When the addition was completed, the mixture was stirred at room

temperature and monitored by ^{31}P NMR. At the end of the reaction, CH_2Cl_2 (5 mL) was added for maximum dissolution of the precipitation. The organic solution was added to a solution of 1 N HCl in diethyl ether (1.5 mmol) followed by an addition of water (5 mL). The organic phase was separated, and the aqueous phase was washed with CH_2Cl_2 (3 × 5 mL). The combined organic phase was dried over $MgSO_4$ and concentrated under reduced pressure. The oil obtained was purified by flash chromatography on silica gel and eluted with a gradient of 1–5% methanol in chloroform, unless other eluent was specified.

Ethyl 3-hydroxypropyl(methyl)phosphinate (4a):

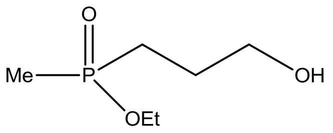

Yield 68%

^{1}H ($CDCl_3$, 300 MHz) δ 4.88 (bs, OH), 4.10 (dq, J_{H-H}= 7.2, J_{P-H}= 2.2 Hz, 2H), 3.74 (t, J_{H-H}= 4.8 Hz, 2H), 1.98-1.78 (m, 4H), 1.51 (d, J_{P-H}= 13.8 Hz, 3H), 1.35 (t, J_{H-H}= 7.2 Hz, 3H); ^{13}C ($CDCl_3$ 75.5 MHz) δ 62.59 (d, J_{P-C}= 10.6 Hz), 60.37 (d, J_{P-C}= 6.4 Hz), 27.19 (d, J_{P-C}= 94.1 Hz), 25.47 (d, J_{P-C}= 4.1Hz), 16.63 (d, J_{P-C}= 5.9 Hz), 13.88 (d, J_{P-C}= 92.1 Hz); ^{31}P {^{1}H}NMR ($CDCl_3$, 121.5 MHz) δ +53.5.

GCMS-CI (m/z) 167 (91%) $(M+1)^+$, 136 (16%) (EtOP(O)(Me)(Et)), 121 (100%) (M-OEt).

Ethyl 3-hydroxypropyl(ethyl)phosphinate (4b):

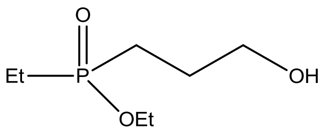

Yield 62%

^{1}H ($CDCl_3$, 500 MHz) δ 4.07 (m, 2H), 3.70 (m, 2H), 1.9-1.71 (m, 6H), 1.31 (t, J_{H-H}= 7.0 Hz, 3H), 1.16 (dt, J_{P-H}= 18.0 Hz, J_{H-H}= 7.5 Hz, 3H); ^{13}C ($CDCl_3$ 125.7 MHz) δ 62.46 (d, J_{P-C}= 10.6 Hz), 60.32 (d, J_{P-C}= 6.5 Hz), 25.13 (d, J_{P-C}= 4.3 Hz), 24.54 (d, J_{P-C}= 90.6 Hz), 21.02 (d, J_{P-C}= 91.7 Hz), 16.52 (d, J_{P-C}= 5.5 Hz), 5.91(d, J_{P-C}= 5.2 Hz); ^{31}P NMR {^{1}H}($CDCl_3$, 202.5 MHz) δ +57.3.

GCMS-CI (m/z) 181 (5.5%) $(M+1)^+$, 106 (91%) (M-(Et+OEt))

Ethyl 3-hydroxypropyl(allyl)phosphinate (4c):

Four equiv of allylmagnesium bromide were used in this reaction.

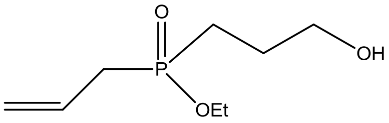

Yield 50%

^{1}H ($CDCl_3$, 500 MHz) δ 5.81-5.74 (m, 1H), 5.23 (d, $J_{H\text{-}H}$= 1.5 Hz, 2H), 4.10 (m, 2H), 3.71 (m, 2H), 2.64 (dd, $J_{H\text{-}P}$= 17.0 Hz, $J_{H\text{-}H}$= 9.0 Hz, 2H), 1.85 (m, 4H), 1.70 (bs, OH), 1.32 (t, $J_{H\text{-}H}$= 7.0 Hz, 3H); ^{13}C ($CDCl_3$ 125.7 MHz) δ 127.70 ($d_{P\text{-}C}$, J= 8.8 Hz), 120.16 (d, $J_{P\text{-}C}$= 12.3 Hz), 62.54 (d, $J_{P\text{-}C}$= 6.4 Hz), 60.64 (d, $J_{P\text{-}C}$= 6.6 Hz), 34.36 (d, $J_{P\text{-}C}$= 86.7 Hz), 24.47 (d, $J_{P\text{-}C}$= 93.2 Hz), 25.08(d, $J_{P\text{-}C}$= 4.5 Hz) 16.54 (d, $J_{P\text{-}C}$= 5.6 Hz); ^{31}P {^{1}H}NMR ($CDCl_3$, 202.5 Mz) δ +51.8.

GCMS-CI (m/z) 193 (40%) $(M+1)^+$, 147 (65%), 123 (100%).

Ethyl 3-hydroxypropyl(cyclopentyl)phosphinate (4d):

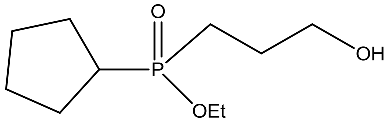

The product was purified using 20% hexane in EtOAc as eluent.

Yield 30%

^{1}H ($CDCl_3$, 300 MHz) δ 4.12 (p, $J_{H\text{-}H}$= 7.2 Hz, 2H), 3.74 (m, 2H), 2.15 (m, 1H), 2.03-1.53 (m, 12H), 1.32 (t, $J_{H\text{-}H}$= 7.2 Hz, 3H); ^{13}C ($CDCl_3$ 75.5 MHz) δ 62.70 (d, $J_{P\text{-}C}$= 9.9 Hz), 60.78 (d, $J_{P\text{-}C}$= 6.8 Hz), 37.37 (d, $J_{P\text{-}C}$= 95.9 Hz), 26.51, 26.40, 25.29 (d, $J_{P\text{-}C}$= 4.6 Hz), 24.62 (d, $J_{P\text{-}C}$= 88.2 Hz), 16.04 (d, $J_{P\text{-}C}$= 5.4 Hz); ^{31}P {^{1}H}NMR ($CDCl_3$, 121.5 MHz) δ +58.4.

GCMS-CI (m/z) 221(100%) $(M+1)^+$, 175 (10%) (M-OEt).

Ethyl 3-hydroxypropyl(phenyl)phosphinate (4e):

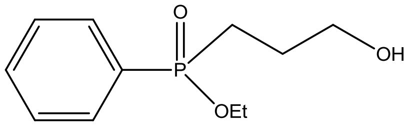

The product was obtained by using only 2 equiv of the Grignard reagent, following the general experimental procedure. The crude oil was purified using 5% methanol in ether as eluent.

Yield 38%

^{1}H ($CDCl_3$, 500 MHz) δ 7.76 (m, 2H), 7.55 (m, 1H), 7.47 (dt, J_{H-H}= 3.5 Hz, J_{P-H}= 7.5 Hz, 2H), 4.00 (dm, J_{P-H}= 11.5 Hz, 2H), 3.71-3.63 (m, 2H), 2.14-2.03 (m, 1H), 2.01-1.90 (m, 1H), 1.88-1.84 (m, 2H), 1.80 (t, J_{H-H}= 7.0 Hz, 3H); ^{13}C ($CDCl_3$ 125.7 MHz) δ 132.1 (d, J_{P-C}= 98.9 Hz), 131.83 (d, J_{P-C}= 2.7 Hz), 130.70 (d, J_{P-C}= 9.5 Hz), 128.60 (d, J_{P-C}= 11.9 Hz), 62.40 (d, J_{P-C}= 8.4 Hz), 30.20, 27.63 (d, J_{P-C}= 71.7 Hz), 25.40 (d, J_{P-C} = 4.3 Hz), 20.40; ^{31}P {^{1}H} NMR ($CDCl_3$, 121.5 MHz) δ +43.1.

GCMS-CI (m/z) 229(5%) $(M+1)^+$, 183 (100%) (M-OEt)

Phenyl(3-hydroxypropyl)(methyl)phosphine oxide (5a):

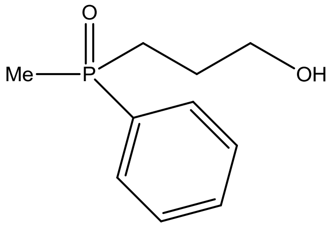

Yield 77%

^{1}H ($CDCl_3$, 500 MHz) δ 7.68 (dd, J_{P-H}= 11.0 Hz, J_{H-H}= 7.5 Hz, 2H), 7.50 (m, 1H), 7.44 (m, 2H), 4.51 (bs, OH), 3.64 (t, J_{H-H}= 5.0 Hz, 2H), 2.15-1.97 (m, 2H), 1.78 (m, 2H), 1.71 (d, J_{P-H}= 13.0 Hz, 3H); ^{13}C ($CDCl_3$ 125.7 MHz) δ 132.59 (d, J_{P-C}= 97.1 Hz), 131.76 (d, J_{P-C}= 2.1 Hz), 129.98 (d, J_{P-C}= 9.3 Hz), 128.67 (d, J_{P-C}= 11.6), 62.03 (d, J_{P-C}= 10.1 Hz), 28.91 (d, J_{P-C}= 70.2 Hz), 25.24 (d, J_{P-C}= 4.2 Hz), 15.81 (d, J_{P-C}= 70.3 Hz); ^{31}P {^{1}H}NMR ($CDCl_3$, 202.5 MHz) δ +37.6

GCMS-CI (m/z) 199 (20%) $(M+1)^+$, 167 (15%) ($M-CH_2OH$), 121 (56%) (M-Ph).

Phenyl(3-hydroxypropyl)(ethyl)phosphine oxide (5b):

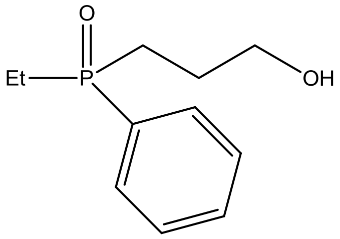

Yield 79%

^{1}H ($CDCl_3$, 500 MHz) δ 7.67(m, 2H), 7.53 (m, 3H), 3.62 (m, 2H), 2.18-2.09 (m, 1H), 2.09-1.98 (m, 2H), 1.95-1.87 (m 1H), 1.87-1.78 (m, 2H), 1.11 (t J_{P-H}= 17.0 Hz, J_{H-H}= 8.0 Hz, 3H); ^{13}C ($CDCl_3$ 125.7 MHz) δ 131.72 (d, J_{P-C}= 2.6 Hz), 131.27 (part of a doublet of the quaternary carbon, the other peak was integrated with the next one), 130.50 (d, J_{P-C}= 8.9 Hz),

128.70 (d, J_{P-C}= 11.4 Hz), 62.40 (d, J_{P-C}= 5.0 Hz), 27.15 (d, J_{P-C}= 67.9 Hz), 25.38 (d, J_{P-C}= 4.8 Hz), 22.66 (d, J_{P-C}= 70.1 Hz), 5.41 (d, J_{P-C}= 5.2 Hz); ^{31}P {^{1}H}NMR ($CDCl_3$, 202.5 MHz) δ +41.9

GCMS-CI (m/z) 213 (100%) $(M+1)^+$, 195 (17%) (M-(OH)).

Allyl(phenyl)(3-hydroxypropyl)phosphine oxide (5c):

Four equiv of allylmagnesium bromide were used in this reaction.

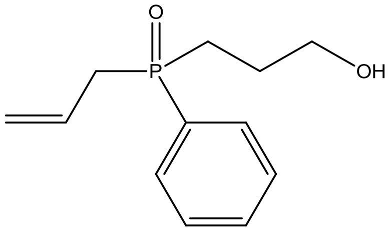

Yield 71%

^{1}H ($CDCl_3$, 500 MHz) δ 7.70 (m, 2H), 7.53 (m, 3H), 5.76 (m, 1H), 5.20 (dd J_{P-H}= 10.3 Hz, J_{H-H}= 3.3 Hz, 1H), 5.15 (dd J_{P-H}= 17.0, Hz, J_{H-H}= 4.8 Hz, 1H), 3.67 (t, J_{H-H}= 5.5 Hz, 2H), 2.85 (dd, J_{P-H}= 10.5 Hz, J_{H-H}= 7.5 Hz, 2H), 2.23-2.15 (m, 1H), 2.12-2.02 (m, 1H), 1.90-1.79 (m, 2H) 1.69 (bs, OH); ^{13}C ($CDCl_3$ 125.7 MHz) δ 131.84 (d, J_{P-C}= 2.9 Hz), 131.02 (d, J_{P-C}= 94.0 Hz), 130.54 (d, J_{P-C}= 8.9 Hz), 128.62 (d, J_{P-C}= 11.4), 127.09 (d, J_{P-C}= 8.9 Hz), 120.73 (d, J_{P-C}= 11.6 Hz), 62.38 (d, J_{P-C}= 7.9 Hz), 36.31 (d, J_{P-C}= 65.7 Hz), 26.57 (d, J_{P-C}= 68.8 Hz), 25.28 (d, J_{P-C}= 4.5 Hz); ^{31}P {^{1}H}NMR ($CDCl_3$, 202.5 MHz) δ +37.2.

GCMS-CI (m/z) 225 (100%) $(M+1)^+$, 207 (27%) $(M-OH)^+$, 183 (32%) (M-(CH_2=$CHCH_2$)).

Phenyl(cyclopentyl)(3-hydroxypropyl)phosphine oxide (5d):

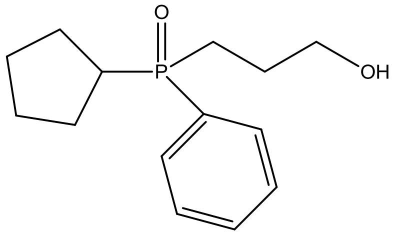

Yield 62%

^{1}H ($CDCl_3$, 500 MHz) δ 7.70 (m, 2H), 7.50 (m, 3H), 4.05 (bs, OH), 3.61 (m, 2H), 2.31 (m, 1H), 2.14 (m, 1H), 2.05 (m, 1H), 1.96 (m, 2H), 1.72-1.60 (m, 8H); ^{13}C ($CDCl_3$ 75.5 MHz) δ 131.62 (d, J_{P-C}= 2.5 Hz), 131.25 (d, J_{P-C}= 108.6 Hz), 130.80 (d, J_{P-C}= 8.4 Hz), 128.66 (d, J_{P-C}= 11.0 Hz), 62.61 (d, J_{P-C}= 6.9 Hz), 38.33 (d, J_{P-C}= 73.1 Hz), 26.83 (d, J_{P-C}= 76.9 Hz),

26.75 (d, $J_{P\text{-}C}$= 9.1 Hz), 26.26 (d, $J_{P\text{-}C}$= 9.6 Hz), 25.61 (d, $J_{P\text{-}C}$= 4.8 Hz); ^{31}P {^{1}H}NMR ($CDCl_3$, 121.5 MHz) δ +42.82.

The quaternary carbon of the phenyl ring was not detected.

GCMS-CI (m/z) 253 (23%) $(M+1)^+$, 235 (12%) (M-OH), 193 (42%) ($PhPO(C_5H_9)$).

Diphenyl(3-hydroxypropyl)phosphine oxide (5e):

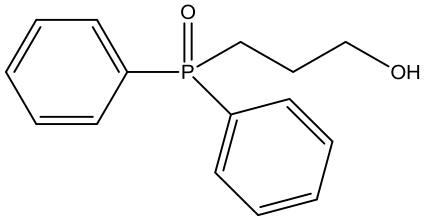

Yield 95%

^{1}H ($CDCl_3$, 500 MHz) δ 7.75 (m, 4H), 7.55-7.50 (m, 2H), 7.49-7.46 (m, 4H), 3.74 (t, $J_{H\text{-}H}$= 5.4 Hz, 2H), 2.45 (dt, $J_{P\text{-}H}$= 11.4 Hz, $J_{H\text{-}H}$= 6.9 Hz, 2H), 1.84 (m, 2H), 1.70 (bs, OH); ^{13}C ($CDCl_3$ 125.7 MHz) δ 132.14 (d, $J_{P\text{-}C}$= 99.5 Hz), 131.83 (d, $J_{P\text{-}C}$= 2.6 Hz), 130.73 (d, $J_{P\text{-}C}$= 9.3 Hz), 128.67 (d, $J_{P\text{-}C}$= 11.7 Hz), 62.41 (d, $J_{P\text{-}C}$= 8.6 Hz), 27.53 (d, $J_{P\text{-}C}$= 71.9 Hz), 25.37 (d, $J_{P\text{-}C}$= 4.2 Hz); ^{31}P {^{1}H}NMR ($CDCl_3$, 121.5 MHz) δ +32.2

GCMS-CI (m/z) 261 (30%) $(M+1)^+$, 243 (43%) (M-OH), 215 (100%) ($Ph_2P(O)CH_2$).

3-(Trimethylsilyl)prop-2-yn-1-yl(phenyl)(3-hydroxypropyl)phosphine oxide (5g):

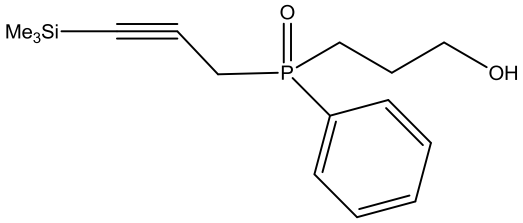

The same general procedure was applied with the following exceptions: 1) THF was used instead of Et_2O. 2) 5 equiv (trimethylsilyl)-propargylmagnesium bromide **7** was used in this reaction. 3) The reaction was refluxed for 18 h before work-up.

Yield 35%

^{1}H ($CDCl_3$, 300 MHz) δ 7.94-7.85 (m, 2H), 7.64-7.48 (m, 3H), 3.75 (t, $J_{H\text{-}H}$= 5.1 Hz, 2H), 3.20-2.90 (m, 2H), 2.50-2.22 (m, 2H), 2.07-1.86 (m, 2H), 1.67 (bs, OH), 0.17 (s, 9H); ^{13}C ($CDCl_3$ 75.5 MHz) δ 132.37 (d, $J_{P\text{-}C}$= 2.7 Hz), 131.07 (d, $J_{P\text{-}C}$= 97.1 Hz), 131.00 (d, $J_{P\text{-}C}$= 8.7 Hz), 128.50 (d, $J_{P\text{-}C}$= 11.7 Hz), 97.12 (d, $J_{P\text{-}C}$= 9.0 Hz), 89.80 (d, $J_{P\text{-}C}$= 7.5 Hz), 62.47 (d, $J_{P\text{-}}$

$_C$= 8.5 Hz), 25.59 (d, J_{P-C}= 70.9 Hz), 25.37 (d, J_{P-C}= 4.5 Hz), 23.81 (d, J_{P-C}= 65.5 Hz), -0.295; ^{31}P {^{1}H}NMR ($CDCl_3$, 121.5 MHz) δ +35.6.

GCMS-CI (m/z) 295 (25%) $(M+1)^+$, 277 (50%) (M-OH), 235 (10%) (M-[$(CH_2)_3$OH]), 183 (92%) (M-(CH_2CCTMS).

Propa-1,2-dien-1-yl(phenyl)(3-hydroxypropyl)phosphine oxide (5f):

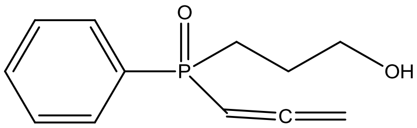

The trimethylsilyl group was cleaved by addition of NH_4F (10 equiv) to a methanolic solution of **5g**. The reaction mixture was stirred at room temperature for 24 h and the solvent was evaporated under vacuum. The precipitation was dissolved in water and the product was extracted with EtOAc, dried over $MgSO_4$ and evaporated under reduced pressure.

^{1}H ($CDCl_3$, 500 MHz) δ 7.72 (m, 2H), 7.57 (m, 3H), 5.65 (dt, J_{H-H}= 6.9 Hz, J_{H-P}= 2.7 Hz, 1H), 5.05 (dd, J_{H-P}= 10.8 Hz, J_{H-H}= 6.9 Hz, 2H), 3.71 (t, J_{H-H}= 5.6 Hz, 2H), 2.36 (m, 2H), 2.15 (m, 2H), 1.83 (bs); ^{13}C ($CDCl_3$ 125.7 MHz) δ 213.36, 132.32 (d, J_{P-C}= 1.9 Hz), 132.08 (d, J_{P-C}= 101.4 Hz), 131.00 (d, J_{P-C}= 9.5 Hz), 128.98 (d, J_{P-C}= 11.7 Hz), 84.47 (d, J_{P-C}= 100.2 Hz), 76.77 (d, J_{P-C}= 12.3 Hz), 62.84 (d, J_{P-C}= 9.0 Hz), 28.60 (d, J_{P-C}= 74.8 Hz), 25.77 (d, J_{P-C}= 4.1 Hz); ^{31}P {^{1}H}NMR ($CDCl_3$, 121.5 MHz) δ +28.3.

GCMS-CI (m/z) 223 (42%) $(M+1)^+$, 183 (63%) (M-$CHCCH_2$), 163 (9%) (M-[$(CH_2)_3$OH]).

^{1}H and ^{13}C NMR charts

4a:

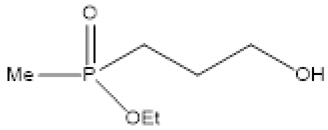

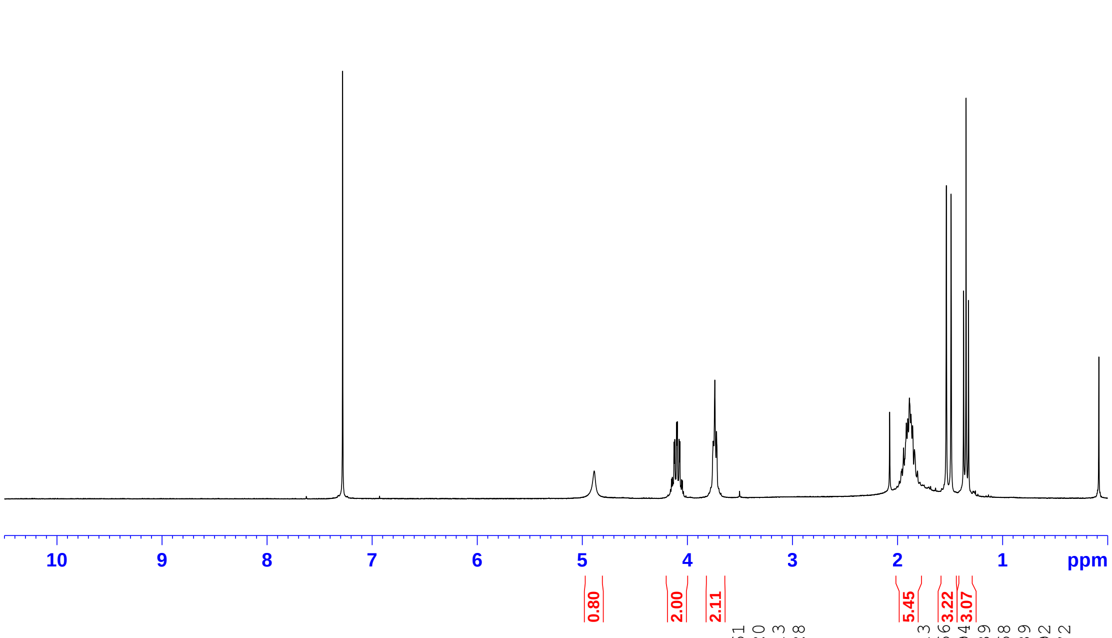

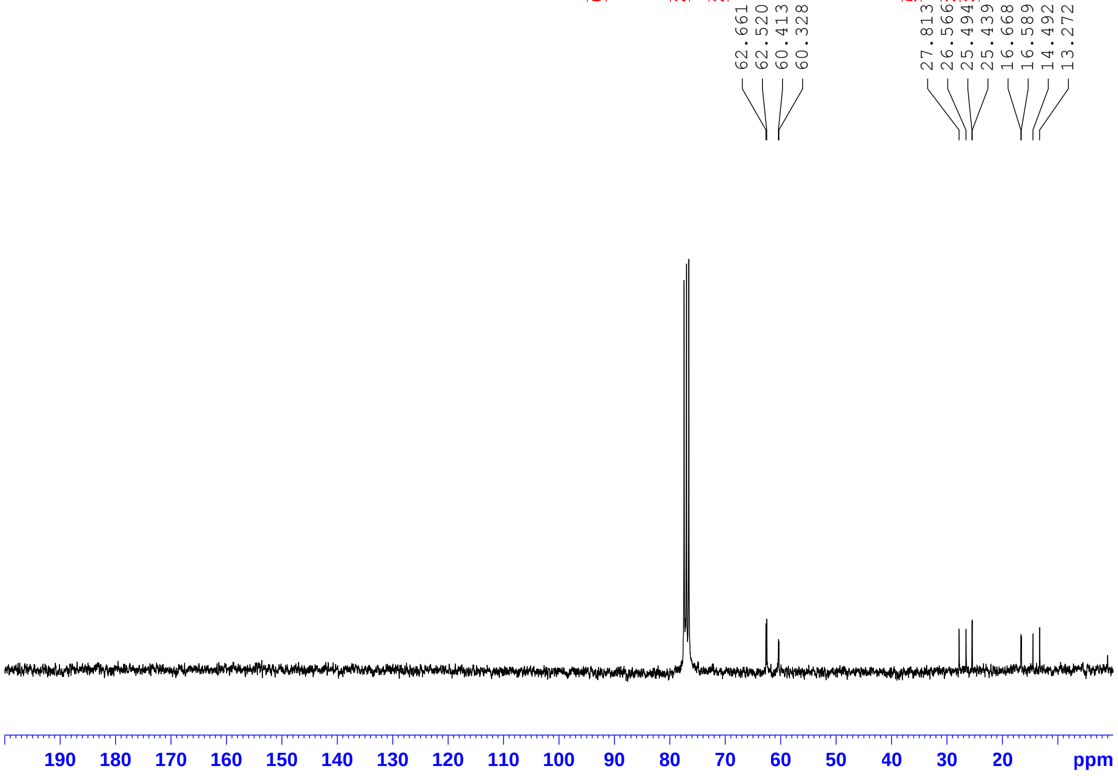

4b:

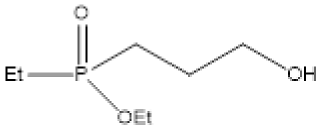
O
Et
P
OH
OEt

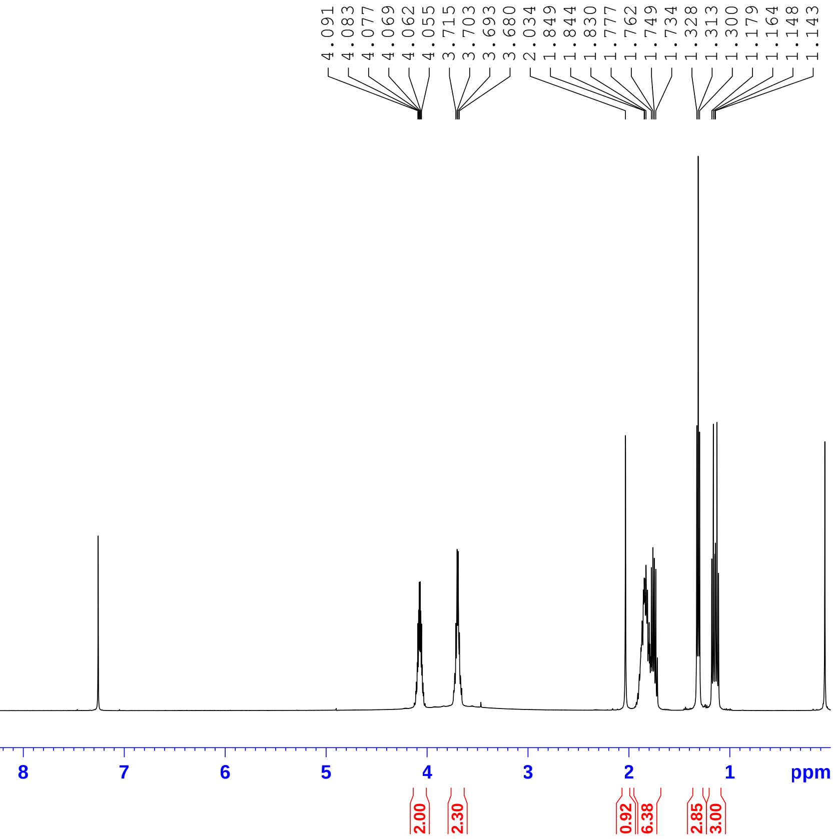
4.091
4.083
4.077
4.069
4.062
4.055
3.715
3.703
3.693
3.680
2.034
1.849
1.844
1.830
1.777
1.762
1.749
1.734
1.328
1.313
1.300
1.179
1.164
1.148
1.143
10
9
8
7
6
5
4
3
2
1
ppm
2.00
2.30
0.92
6.38
2.85
3.00

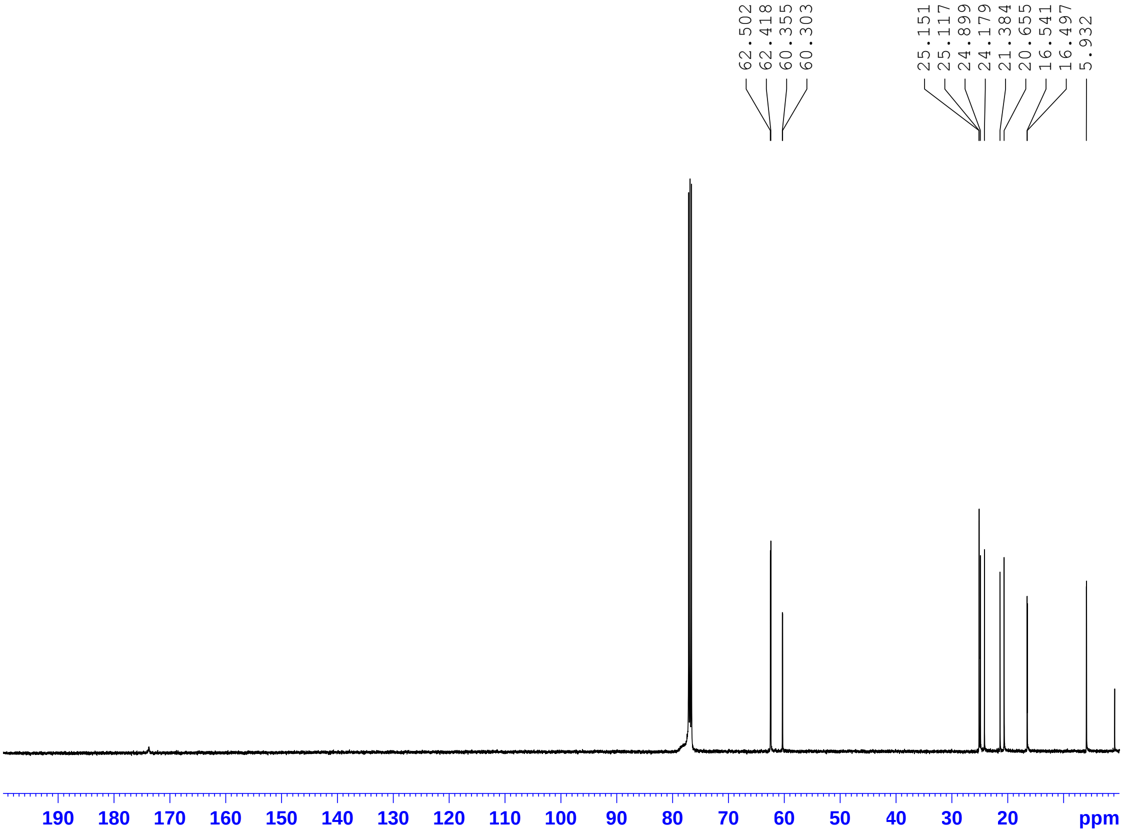
62.502
62.418
60.355
60.303
25.151
25.117
24.899
24.179
21.384
20.655
16.541
16.497
5.932
190
180
170
160
150
140
130
120
110
100
90
80
70
60
50
40
30
20
ppm

4c:

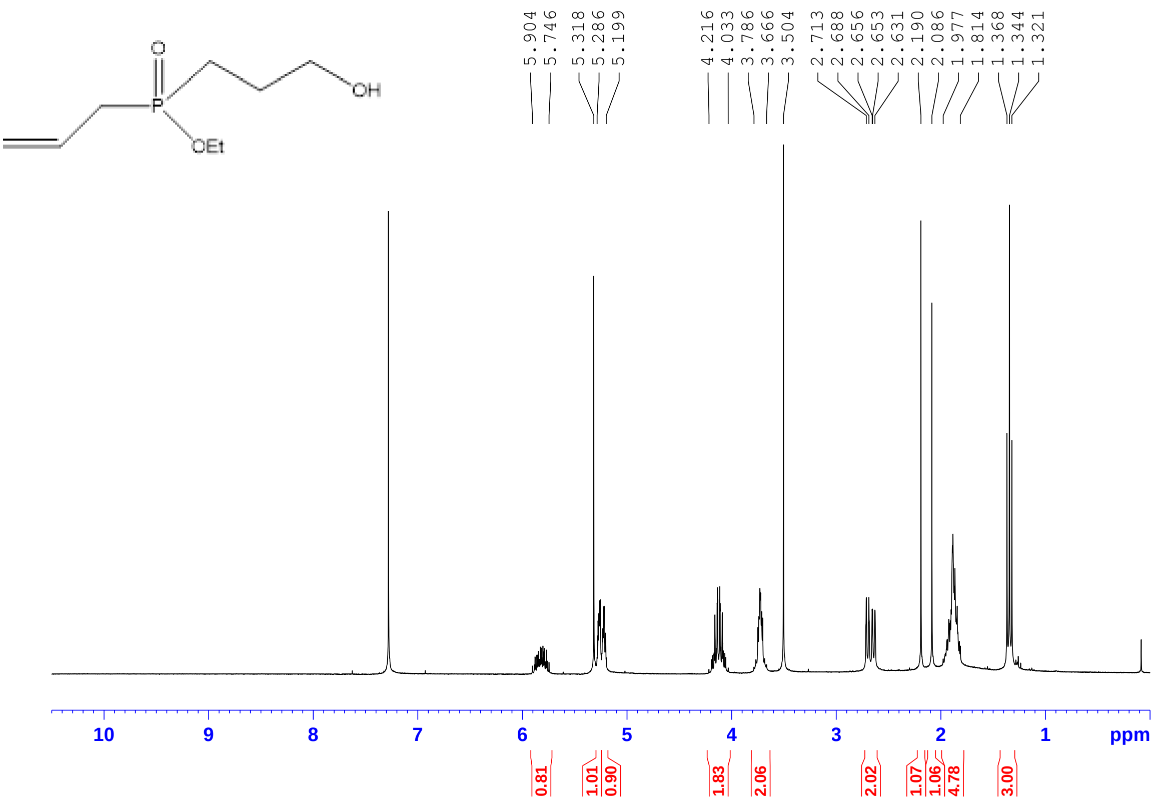
O
P
OH
OEt
5.904
5.746
5.318
5.286
5.199
4.216
4.033
3.786
3.666
3.504
2.713
2.688
2.656
2.653
2.631
2.190
2.086
1.977
1.814
1.368
1.344
1.321
10
9
8
7
6
5
4
3
2
1
ppm
0.81
1.01
0.90
1.83
2.06
2.02
1.07
1.06
4.78
3.00

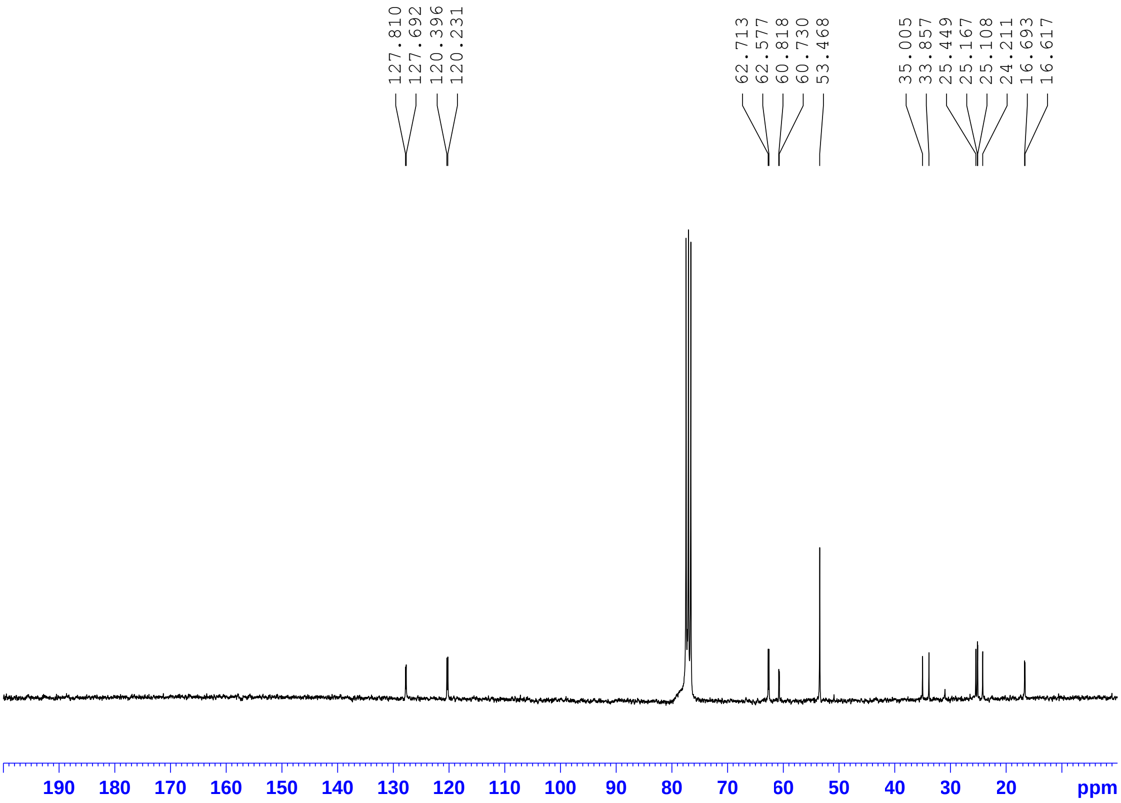
127.810
127.692
120.396
120.231
62.713
62.577
60.818
60.730
53.468
35.005
33.857
25.449
25.167
25.108
24.211
16.693
16.617
190
180
170
160
150
140
130
120
110
100
90
80
70
60
50
40
30
20
ppm

4d:

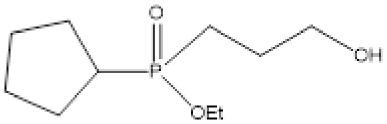
O
P
OEt
OH

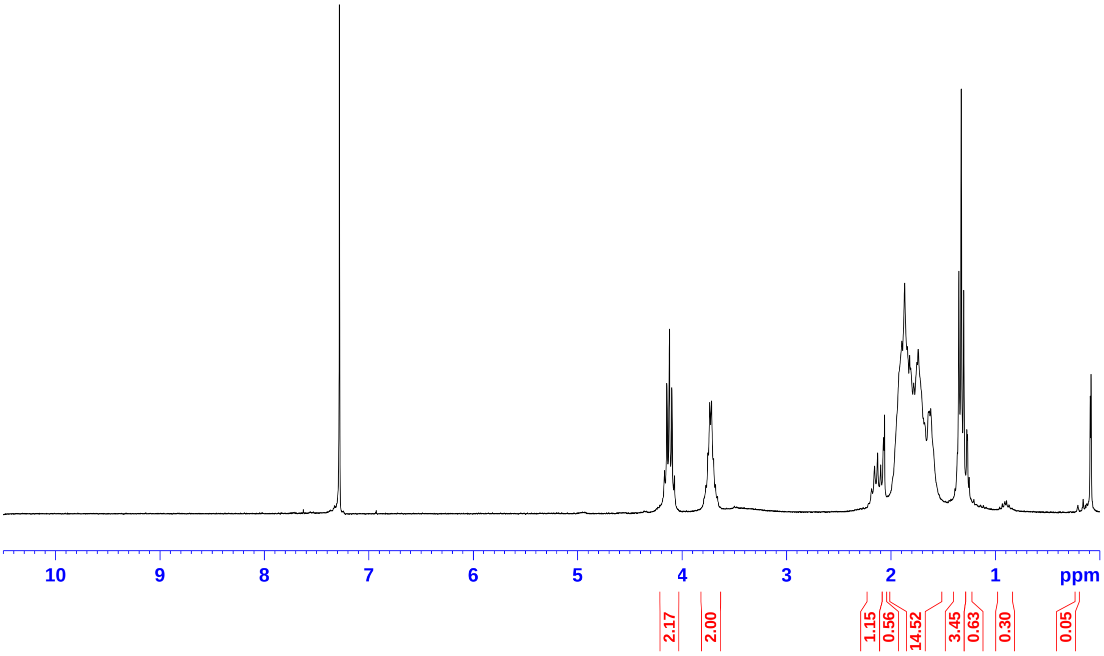
4.170
4.146
4.122
4.098
4.074
3.772
3.753
3.736
3.719
3.682
2.186
2.158
2.129
2.099
2.072
2.063
1.351
1.327
1.304
1.275
1.268
1.251
0.959
0.933
0.910
10
9
8
7
6
5
4
3
2
1
ppm
2.17
2.00
1.15
0.56
14.52
3.45
0.63
0.30
0.05

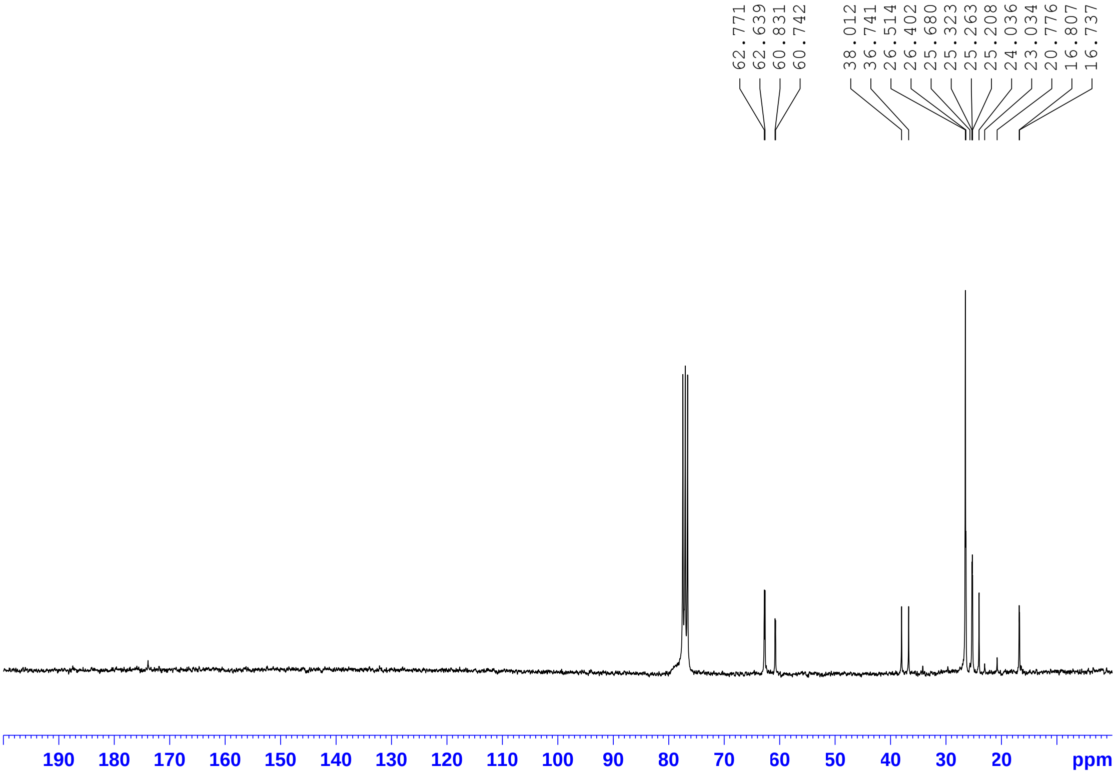
62.771
62.639
60.831
60.742
38.012
36.741
26.514
26.402
25.680
25.323
25.263
25.208
24.036
23.034
20.776
16.807
16.737
190
180
170
160
150
140
130
120
110
100
90
80
70
60
50
40
30
20
ppm

4e:

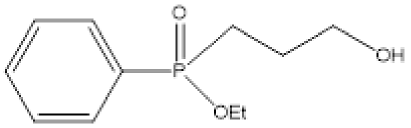
O
P
OH
OEt

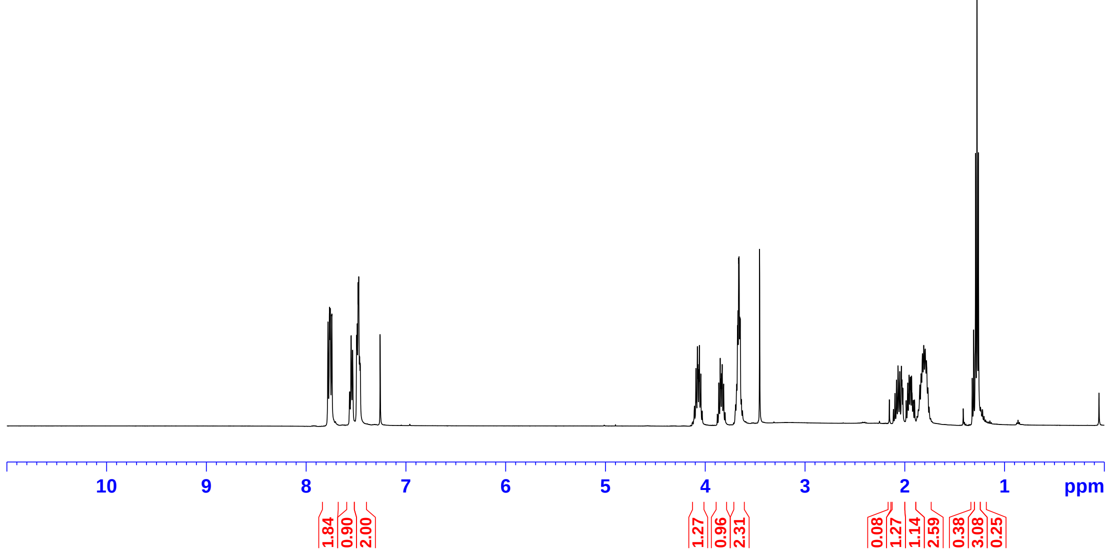
7.782
7.768
7.766
7.759
7.745
7.743
7.549
7.535
7.495
7.488
7.480
7.473
7.465
7.464
4.094
4.079
4.074
4.065
4.059
4.045
3.850
3.836
3.830
3.677
3.673
3.665
3.662
3.654
3.649
2.084
2.068
2.050
2.035
2.028
1.957
1.945
1.932
1.837
1.834
1.823
1.810
1.795
1.783
10
9
8
7
6
5
4
3
2
1
ppm
1.84
0.90
2.00
1.27
0.96
2.31
0.08
1.27
1.14
2.59
0.38
3.08
0.25

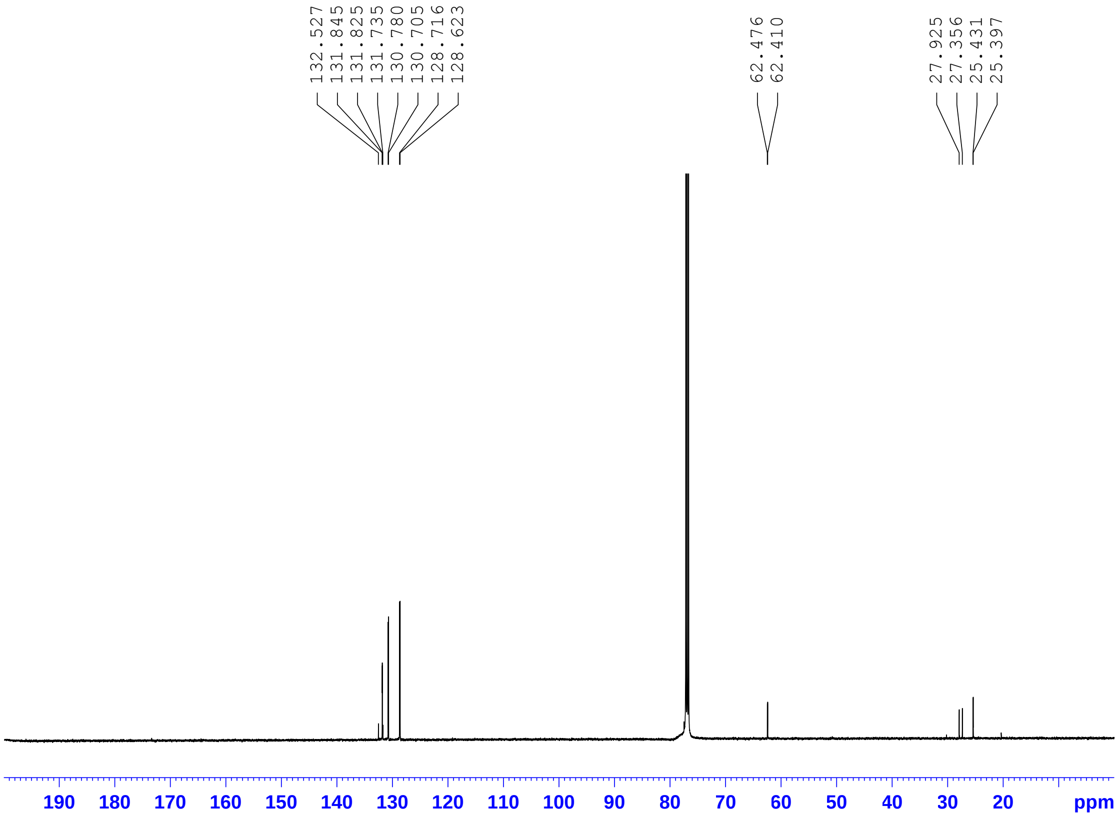
132.527
131.845
131.825
131.735
130.780
130.705
128.716
128.623
62.476
62.410
27.925
27.356
25.431
25.397
190
180
170
160
150
140
130
120
110
100
90
80
70
60
50
40
30
20
ppm

5a:

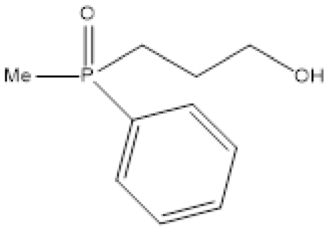

O
Me
P
OH

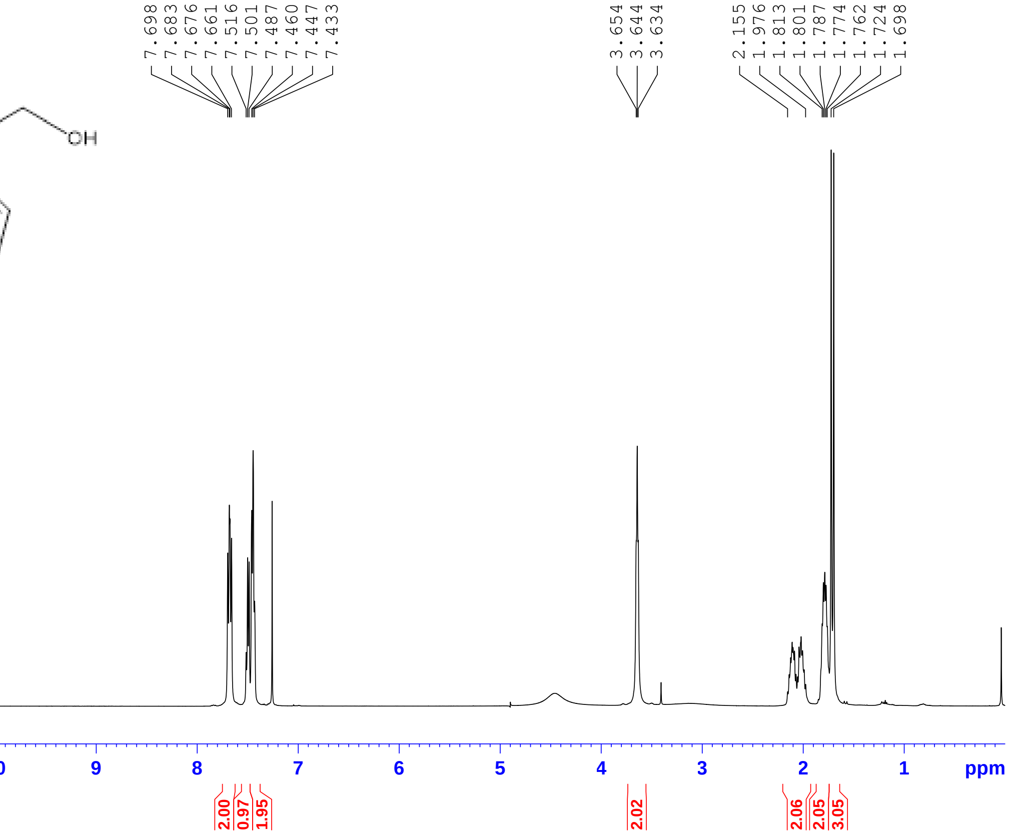

7.698
7.683
7.676
7.661
7.516
7.501
7.487
7.460
7.447
7.433
3.654
3.644
3.634
2.155
1.976
1.813
1.801
1.787
1.774
1.762
1.724
1.698
10
9
8
7
6
5
4
3
2
1
ppm
2.00
0.97
1.95
2.02
2.06
2.05
3.05

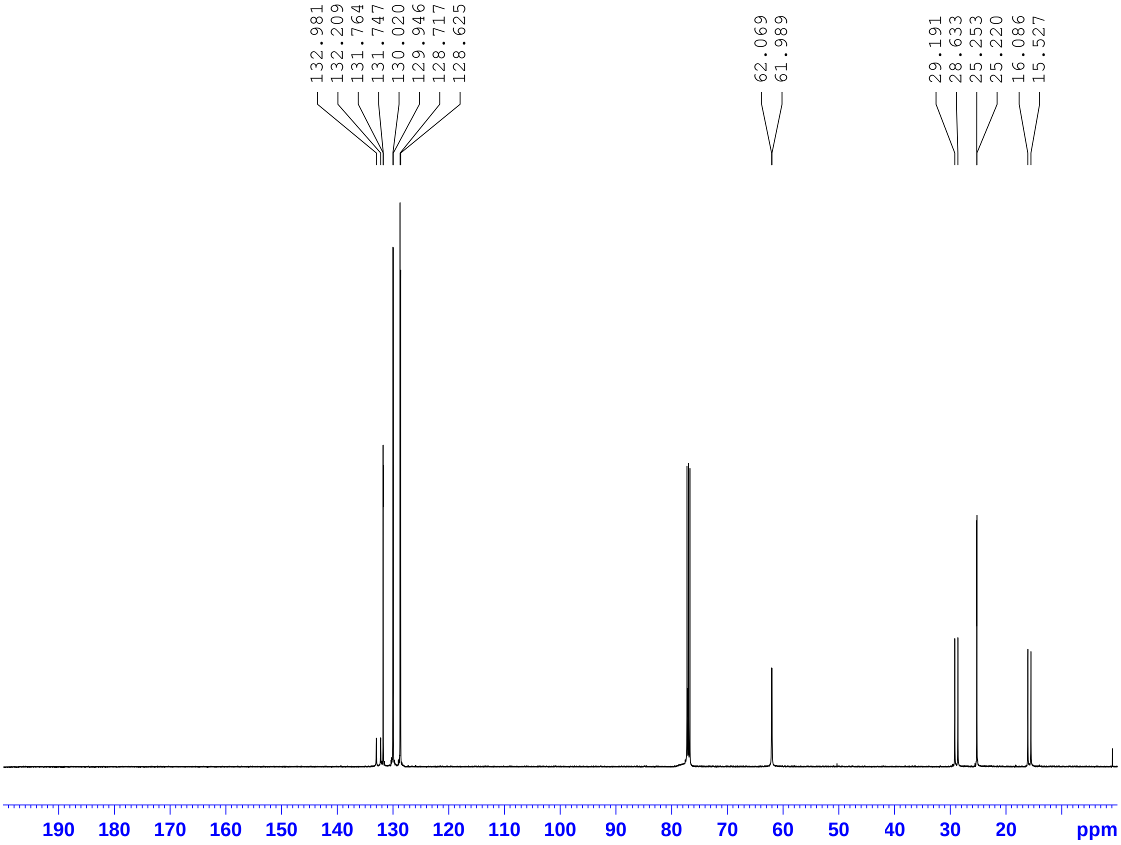

132.981
132.209
131.764
131.747
130.020
129.946
128.717
128.625
62.069
61.989
29.191
28.633
25.253
25.220
16.086
15.527
190
180
170
160
150
140
130
120
110
100
90
80
70
60
50
40
30
20
ppm

5b:

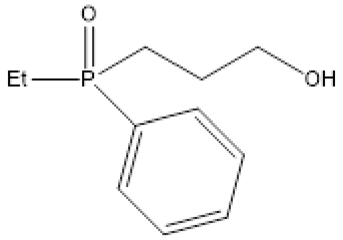
O
Et
P
OH

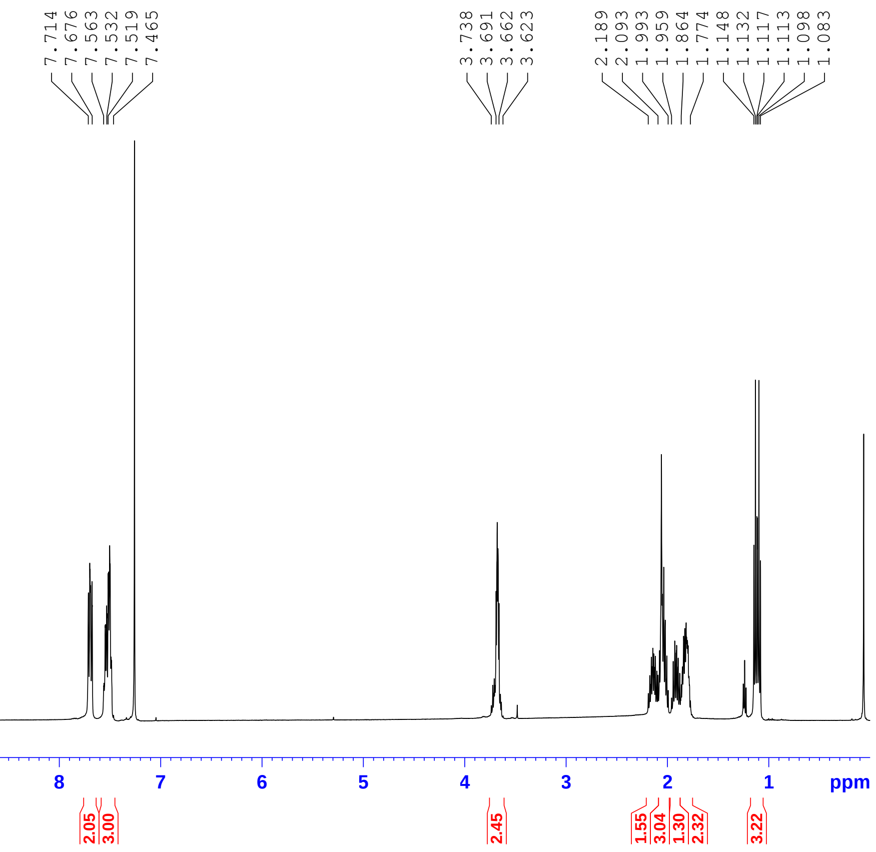
7.714
7.676
7.563
7.532
7.519
7.465
3.738
3.691
3.662
3.623
2.189
2.093
1.993
1.959
1.864
1.774
1.148
1.132
1.117
1.113
1.098
1.083
10
9
8
7
6
5
4
3
2
1
ppm
2.05
3.00
2.45
1.55
3.04
1.30
2.32
3.22

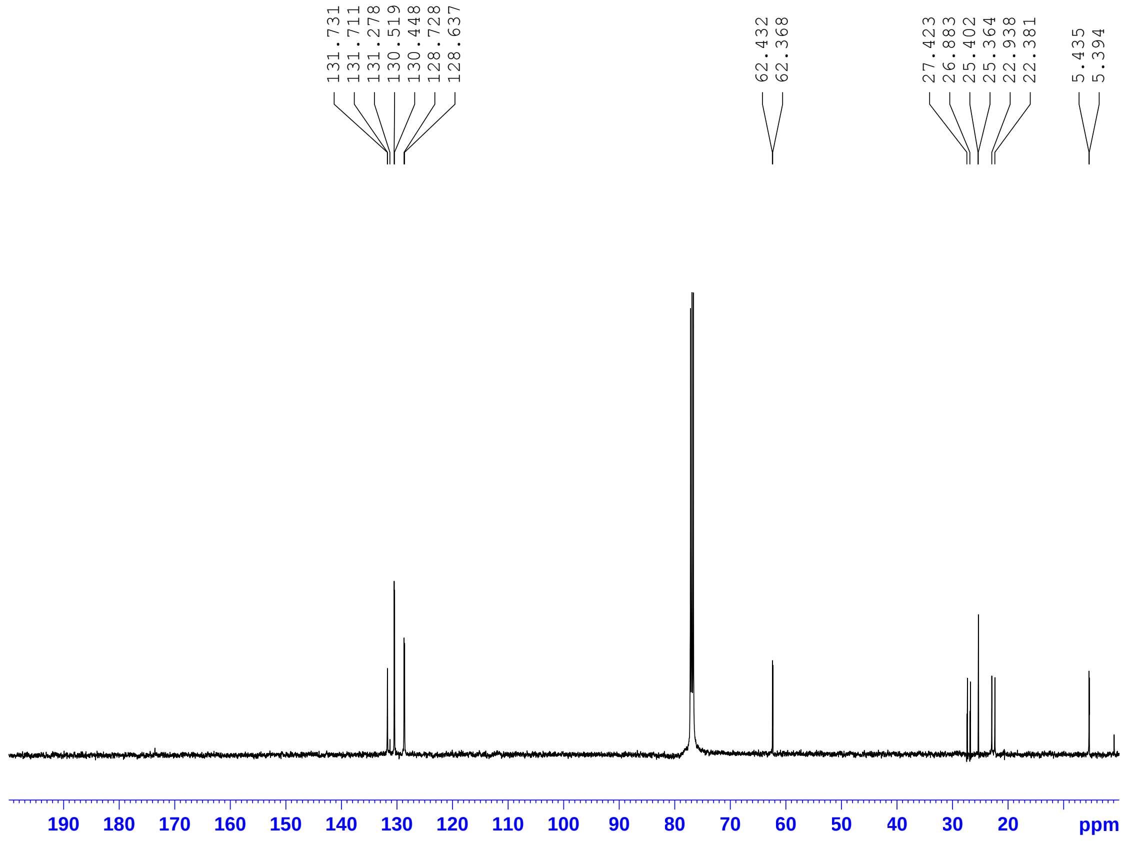
131.731
131.711
131.278
130.519
130.448
128.728
128.637
62.432
62.368
27.423
26.883
25.402
25.364
22.938
22.381
5.435
5.394
190
180
170
160
150
140
130
120
110
100
90
80
70
60
50
40
30
20
ppm

5c:

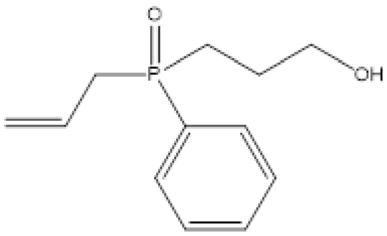
O
P
OH

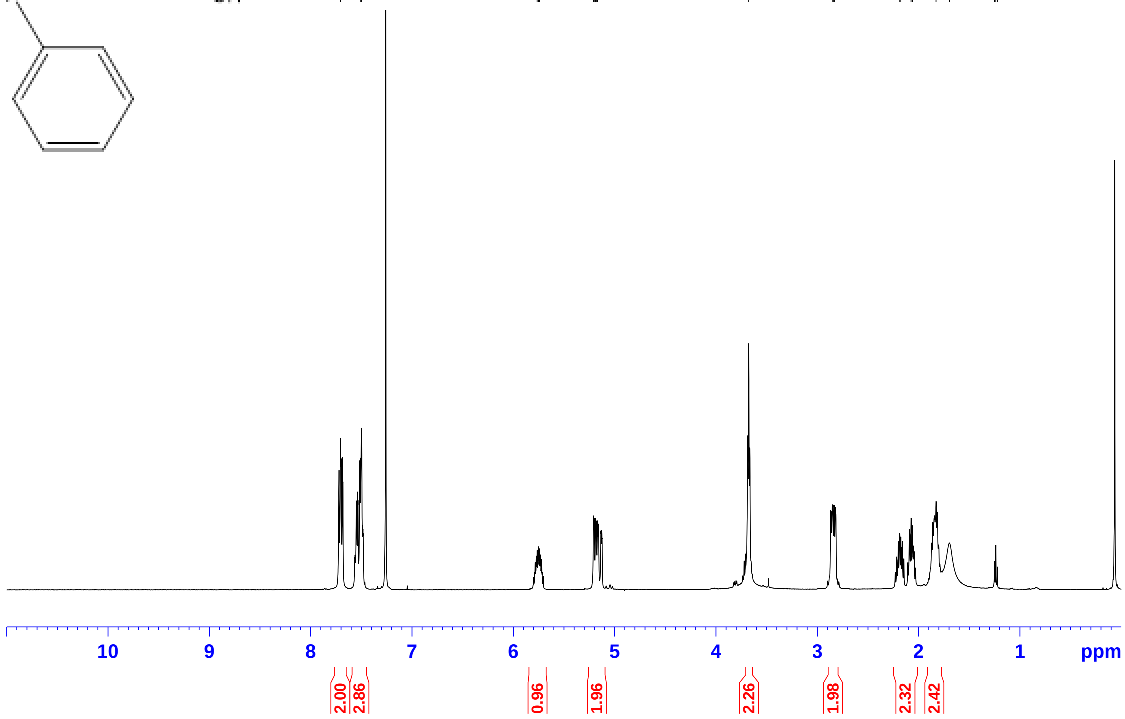
7.707
7.704
7.511
7.501
7.496
5.765
5.755
5.751
5.740
5.208
5.202
5.188
5.181
5.170
5.168
3.677
2.853
2.837
2.835
2.832
2.186
2.176
2.075
2.062
1.828
1.696
1.252
1.238
1.224
10
9
8
7
6
5
4
3
2
1
ppm
2.00
2.86
0.96
1.96
2.26
1.98
2.32
2.42

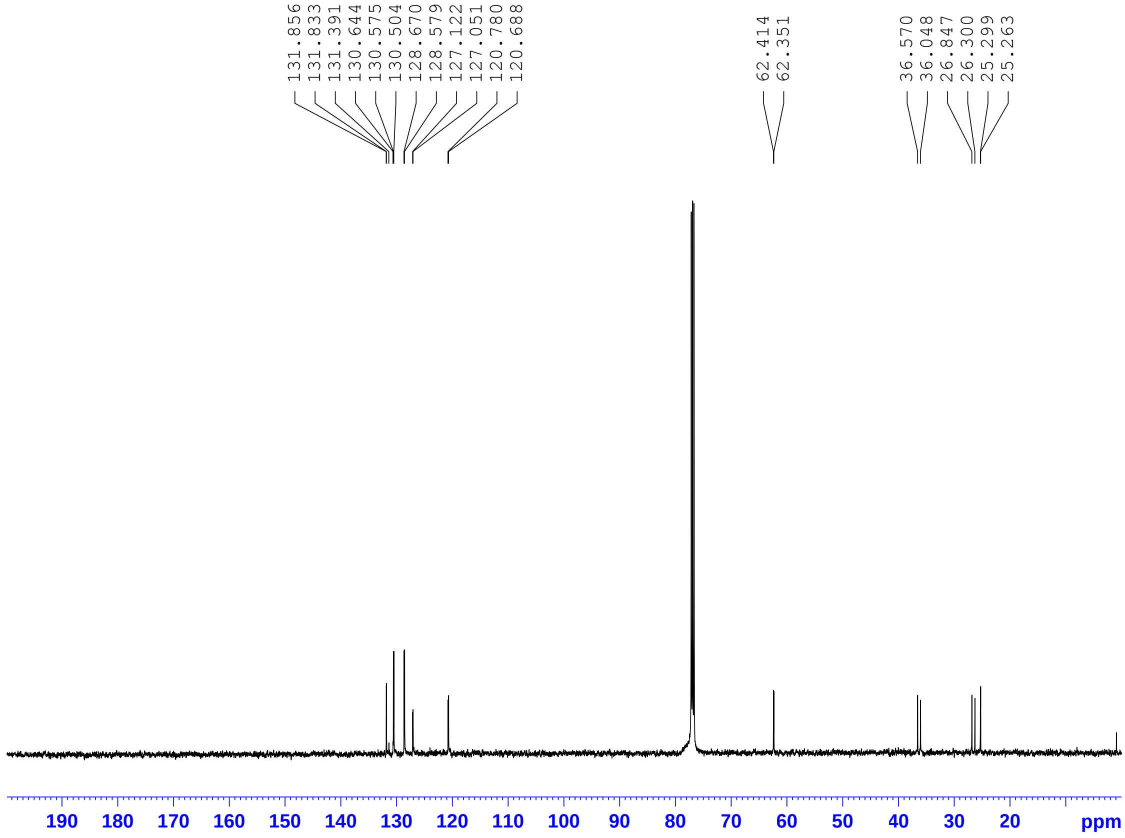
131.856
131.833
131.391
130.644
130.575
130.504
128.670
128.579
127.122
127.051
120.780
120.688
62.414
62.351
36.570
36.048
26.847
26.300
25.299
25.263
190
180
170
160
150
140
130
120
110
100
90
80
70
60
50
40
30
20
ppm

5d:

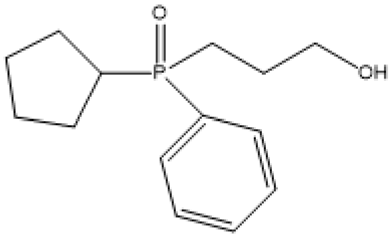
O
P
OH

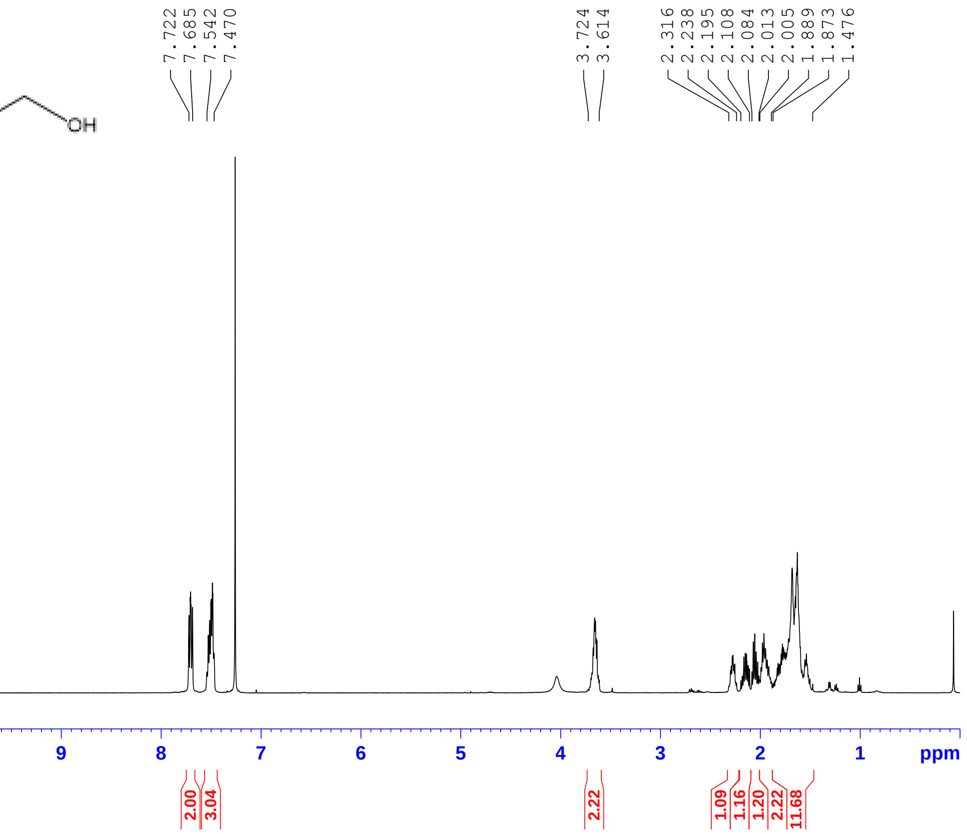
7.722
7.685
7.542
7.470
3.724
3.614
2.316
2.238
2.195
2.108
2.084
2.013
2.005
1.889
1.873
1.476
10
9
8
7
6
5
4
3
2
1
ppm
2.00
3.04
2.22
1.09
1.16
1.20
2.22
11.68

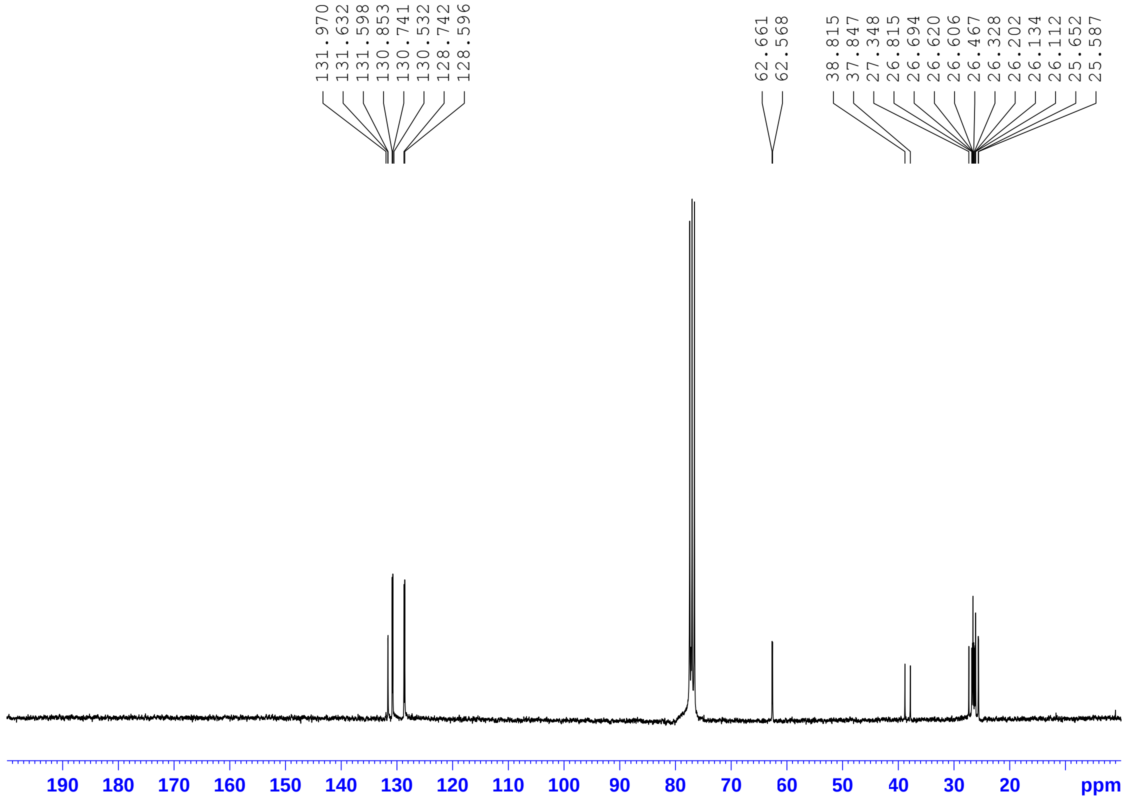
131.970
131.632
131.598
130.853
130.741
130.532
128.742
128.596
62.661
62.568
38.815
37.847
27.348
26.815
26.694
26.620
26.606
26.467
26.328
26.202
26.134
26.112
25.652
25.587
190
180
170
160
150
140
130
120
110
100
90
80
70
60
50
40
30
20
ppm

5e:

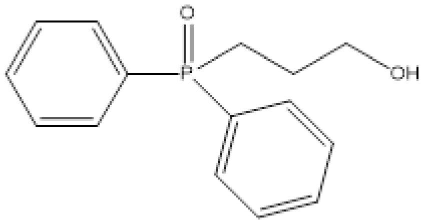
O
P
OH

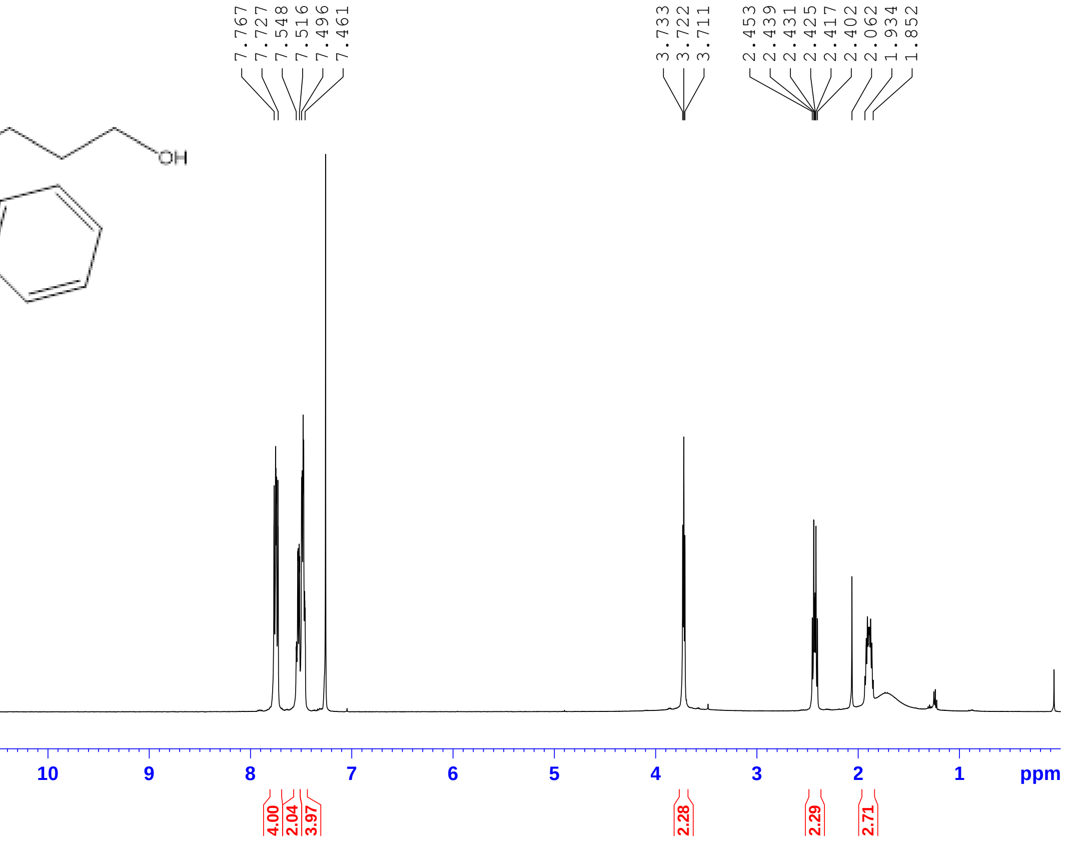
7.767
7.727
7.548
7.516
7.496
7.461
3.733
3.722
3.711
2.453
2.439
2.431
2.425
2.417
2.402
2.062
1.934
1.852
10
9
8
7
6
5
4
3
2
1
ppm
4.00
2.04
3.97
2.28
2.29
2.71

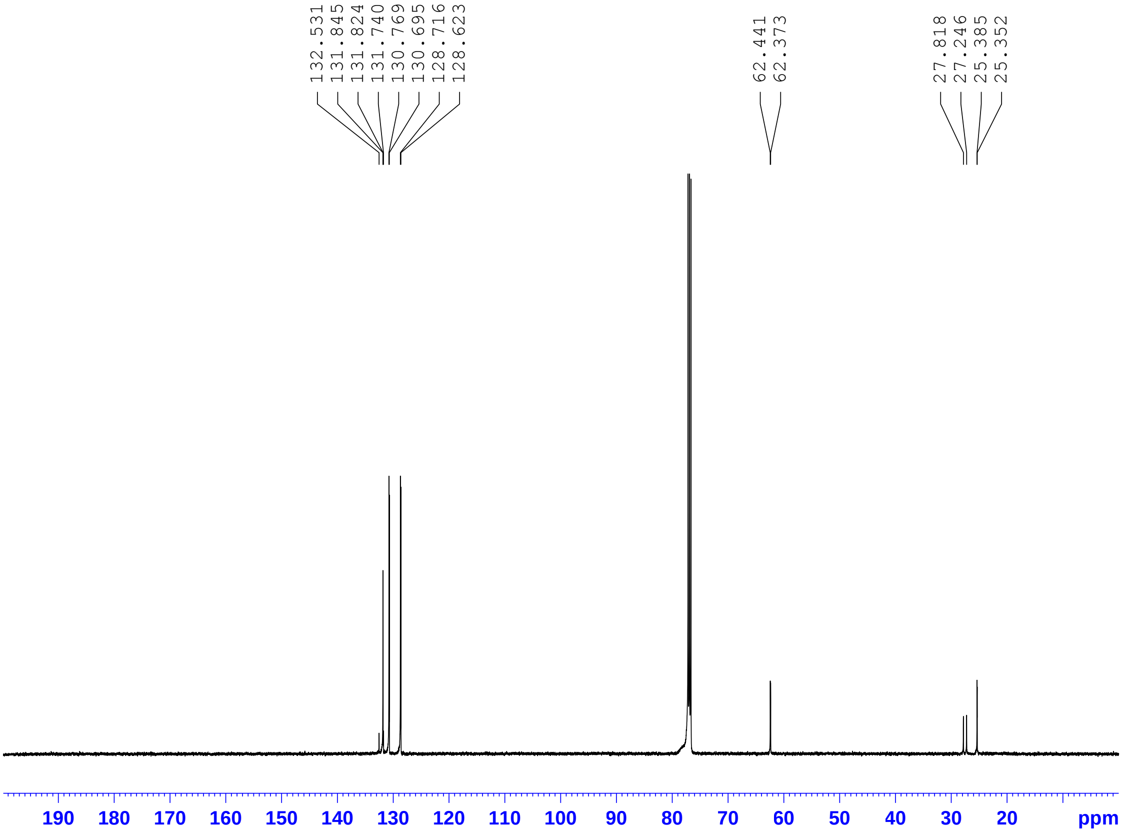
132.531
131.845
131.824
131.740
130.769
130.695
128.716
128.623
62.441
62.373
27.818
27.246
25.385
25.352
190
180
170
160
150
140
130
120
110
100
90
80
70
60
50
40
30
20
ppm

5g:

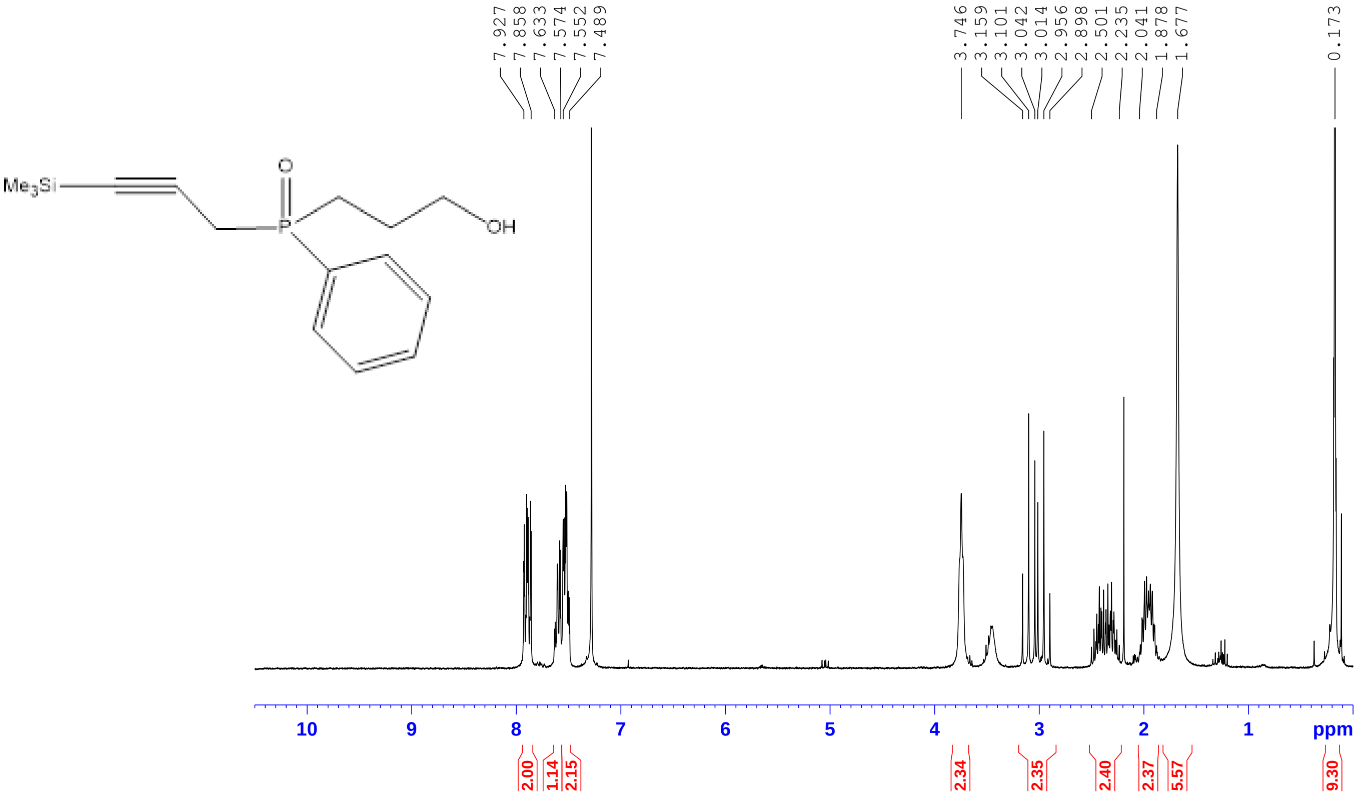

Me3Si
O
P
OH
7.927
7.858
7.633
7.574
7.552
7.489
3.746
3.159
3.101
3.042
3.014
2.956
2.898
2.501
2.235
2.041
1.878
1.677
0.173
10 9 8 7 6 5 4 3 2 1 ppm
2.00
1.14
2.15
2.34
2.35
2.40
2.37
5.57
9.30

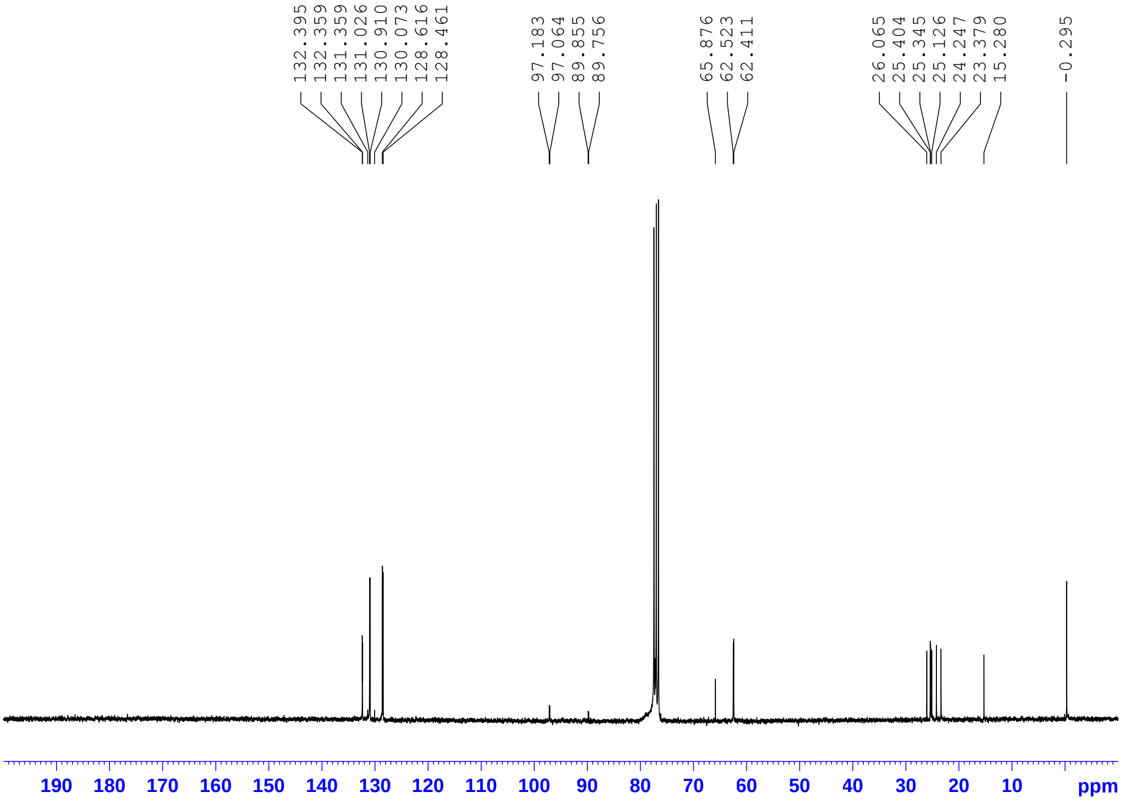

132.395
132.359
131.359
131.026
130.910
130.073
128.616
128.461
97.183
97.064
89.855
89.756
65.876
62.523
62.411
26.065
25.404
25.345
25.126
24.247
23.379
15.280
-0.295
190 180 170 160 150 140 130 120 110 100 90 80 70 60 50 40 30 20 10 ppm

References

1. Vernekar, S. K. V.; Kipke, P.; Redlich, H. *J. Carbohyd. Chem.* **2008**, *27*, 10-31.

2. Phosphonic Acid Phostones", U. S. Patent 2,953,591 20,09,1960.

3. Kluger, R.; Taylor, S. D. *J. Am. Chem. Soc.* **1990**, *112*, 6669-6671.

4. Taylor, S. D.; Kluger, R. *J. Am. Chem. Soc.* **1992**, *114*, 3067-3071.
